# Effect of hydroxychloroquine and characterization of autophagy in a mouse model of endometriosis

**DOI:** 10.1038/cddis.2015.361

**Published:** 2016-01-14

**Authors:** A Ruiz, S Rockfield, N Taran, E Haller, R W Engelman, I Flores, P Panina-Bordignon, M Nanjundan

**Affiliations:** 1 Department of Cell Biology, Microbiology, and Molecular Biology, University of South Florida, Tampa, FL, USA; 2 Department of Integrative Biology, University of South Florida, Tampa, FL, USA; 3 Department of Pathology and Cell Biology, University of South Florida, Tampa, FL, USA; 4 Department of Basic Science-Microbiology, Ponce Health Sciences University and School of Medicine, Ponce Research Institute, Ponce, Puerto Rico; 5 Department of Clinical Sciences and Ob-Gyn, Ponce Health Sciences University and School of Medicine, Ponce Research Institute, Ponce, Puerto Rico; 6 Reproductive Sciences Laboratory, Division of Genetics and Cell Biology, IRCCS Ospedale San Raffaele, Milan, Italy

## Abstract

In endometriosis, the increased survival potential of shed endometrial cells (which normally undergo anoikis) is suggested to promote lesion development. One mechanism that may alter anoikis is autophagy. Using an autophagic flux inhibitor hydroxychloroquine (HCQ), we identified that it reduces the *in vitro* survival capacity of human endometriotic and endometrial T-HESC cells. We also identified that HCQ could decrease lesion numbers and disrupt lesion histopathology, as well as increase the levels of peritoneal macrophages and the IP-10 (10 kDa interferon-*γ*-induced protein) chemokine in a mouse model of endometriosis. We noted that RNA levels of a subset of autophagic markers were reduced in lesions relative to uterine horns from endometriosis-induced (untreated) mice. In addition, the RNA levels of autophagic markers were decreased in uterine horns of endometriosis-induced mice compared with those from controls. However, we noted that protein expression of LC3B (microtubule-associated protein 1 light-chain 3*β*; an autophagic marker) was increased in uterine horns of endometriosis-induced mice compared with uterine horns of controls. By immunohistochemical staining of a human endometriosis-focused tissue microarray, we observed LC3B expression predominantly in epithelial relative to stromal cells in both eutopic and ectopic endometria. Via transmission electron microscopy, cells from eutopic endometria of endometriosis-induced mice contained more lipid droplets (rather than autophagosomes) compared with uterine horns from controls. Collectively, our findings indicate that the autophagic pathway is dysregulated in both ectopic and eutopic endometrium in a murine model of endometriosis and that HCQ has potential as a therapeutic agent for women afflicted with endometriosis.

Endometriosis is a chronic, painful, and debilitating disease in which endometrium-like glandular and stromal cells grow outside the uterine cavity.^1, 2^ It is an inflammatory and estrogen-dependent disease that affects 6–10% of women during their reproductive years and up to 50% of women receiving fertility treatments.^3^ Sampson's hypothesis (the most accepted theory) states that shed endometrial tissue during menses reaches the peritoneal cavity by exiting the uterus through the fallopian tubes by retrograde menstruation.^4, 5, 6^ These shed endometrial cells survive, implant, and grow at ectopic locations, developing into endometriotic lesions.^5, 7^

Epithelial cells normally undergo anoikis, a mechanism of programmed cell death, upon detachment from the extracellular matrix.^8^ One mechanism that we propose could potentially alter the anoikis response in endometrial cells is autophagy. This cellular pathway needs to be carefully regulated to maintain cellular homeostasis.^9, 10^ Under conditions of stress, changes in autophagic flux can lead to altered cellular survival.^9, 10^ Autophagy is a complex process that begins with the formation of double-membrane vesicles, termed autophagosomes, which engulf cytoplasmic components. For a comprehensive review of the autophagic pathway, refer to Feng *et al.*^10^ Briefly, autophagosomes fuse with lysosomes to degrade and recycle their cargo comprised of oxidized proteins, lipids, and damaged organelles. Presently, there is limited evidence that autophagy contributes to the development and progression of endometriosis. In a surgical induction model of murine endometriosis, increased expression of ATG9A, an autophagic mediator that is involved in vesicle formation,^11^ was detected in the eutopic endometria from endometriosis-induced mice.^12^ In human endometriomas (ovarian endometriosis), there was a reduction of LC3-II (the conjugated form of LC3) protein compared with control eutopic endometrial tissue.^13^ In contrast, an independent study reported that the protein expression of LC3-II was elevated, while p62 (which binds ubiquitinated cargo for degradation) was decreased in ovarian endometriomas compared with eutopic endometria of disease-free participants.^14^

Herein, our main aim was to provide further evidence for a role of autophagy in endometriosis development. Specifically, we sought to determine the therapeutic effects of a lysosomotropic agent and known autophagic flux inhibitor, hydroxychloroquine (HCQ),^15, 16, 17^ on human endometriotic cells and in an established mouse model of endometriosis. The results presented herein are of high clinical translational value, as we identify a potential new non-hormonal treatment for this still incurable and common disease.

## Results

### HCQ alters human endometrial and endometriotic cell survival as well as lesion number and histopathology in a mouse model of endometriosis

**Figure 1:**
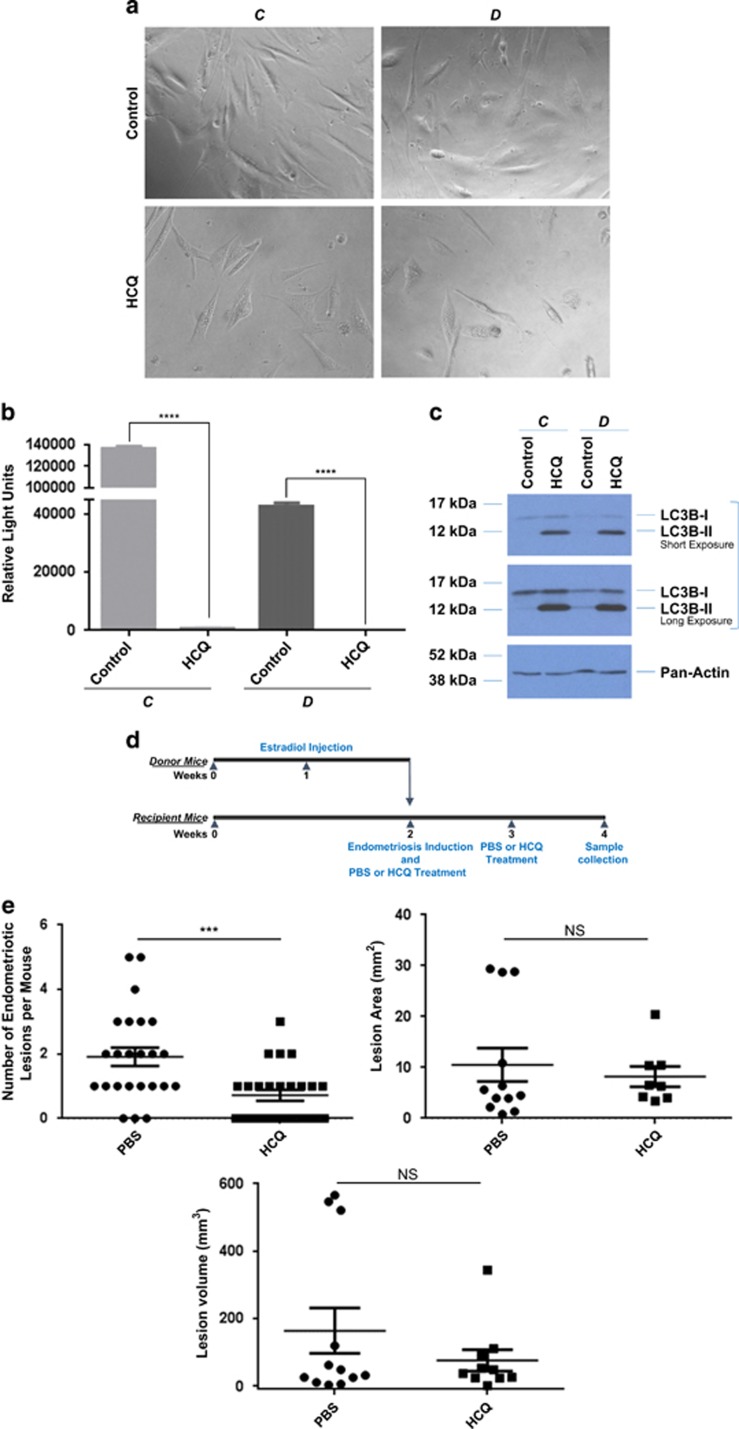

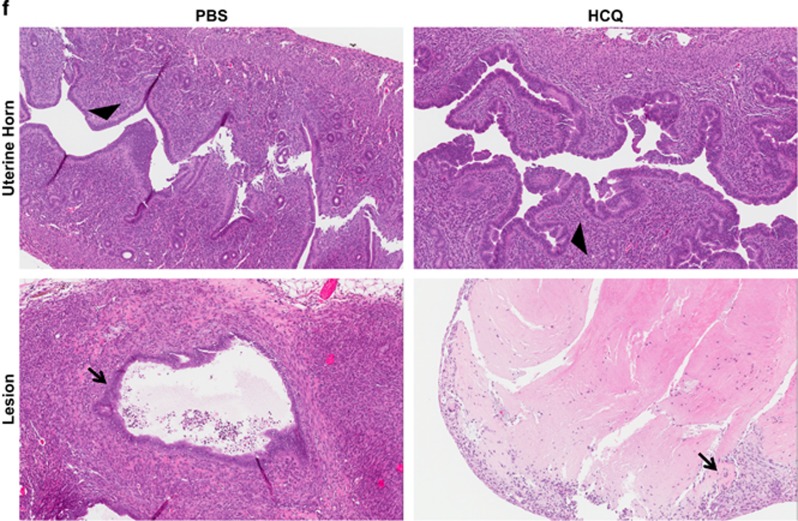
HCQ reduces endometriotic cell survival as well as lesion number and histopathology in a mouse model of endometriosis. (**a**) Representative images of life-extended endometriotic cells using human endometriotic cells derived from two different lesion types: ‘C' and ‘D' treated for 18 h with 25 *μ*M HCQ. (**b**) Cell survival of life-extended endometriotic cells treated with 25 *μ*M HCQ for 5 days was assessed by CellTiter-glo and measuring luminescence. (**c**) Cell lysate from life-extended endometriotic cells treated with 25 *μ*M HCQ for 18 h were analyzed by western blotting using the indicated antibodies. Three independent experiments were performed. (**d**) Schematic representation of the experimental design. Mice were intraperitoneally injected with β-estradiol at 6 weeks of age. After 1 week, these mice were killed and their uterine horns were removed, minced, and injected into the peritoneal cavity of the same-age mice (recipients at 7 weeks of age). The same day of endometriosis induction, mice received an intraperitoneal injection of HCQ or PBS. A second dose was administered 1 week later. Two weeks after induction, mice were killed and samples were collected. (**e**) The lesion numbers, area, and volume per mouse are shown for HCQ- and PBS-treated endometriosis-induced mice. A subset of lesions (PBS- (*n*=12) and HCQ- (*n*=10) treated mice) was measured lengthwise and widthwise to determine the area and volume. (**f**) Uterine horns and lesions from PBS- and HCQ-treated mice were subjected to H&E staining. Black arrowheads indicate glandular compartments (top panels). Black arrows indicate epithelial cells (bottom panels). All images were captured at 10 × magnification

To assess whether an autophagic flux inhibitor could alter the survival capacity of cells isolated from human endometriotic lesions, we treated life-extended human endometriotic cells (cells were derived from peritoneal (‘C') and ovarian (‘D') lesions obtained from two independent patients and were thus tested separately) with 25 *μ*M HCQ. This dose was selected based on our previous studies.^18^ As shown in Figures 1a and b, we observed a marked reduction in cell survival of human endometriotic cells from two different types of lesions (*P*<0.0001) following 5 days of HCQ treatment. To validate the activity of HCQ, we performed western blot analysis for LC3B, which showed that LC3B-II increased with HCQ treatment in these human endometriotic cells (Figure 1c). A similar reduction in cell survival and increase in LC3B-II protein was noted in the T-HESC human endometrial stromal cells (derived from myoma, Supplementary Figure 1). To confirm the effect of autophagy inhibition, we performed siRNA knockdown for ATG5, beclin-1, ATG7, PIK3C3 (phosphatidylinositol 3-kinase, catalytic subunit type 3), and LC3B in our human endometrial and endometriotic cells. Although we failed to obtain suitable numbers of viable cells upon transfection of human endometriotic cells for further analysis, we successfully obtained >90% knockdown efficiency of the above-described autophagic mediators in T-HESC cells (Supplementary Figure 1d). Interestingly, protein levels of p21 (a cyclin-dependent kinase inhibitor involved in cell cycle arrest) markedly increased with siRNA targeting ATG7 and to a lesser degree with LC3B and beclin-1. Therefore, we selected these autophagic mediators to investigate their effects on cell viability using the CellTiter-glo assay in these cells. As shown in Supplementary Figure 1e, the cell viability of T-HESC was particularly reduced with ATG7 knockdown (and to a lesser degree with LC3B and only slightly with beclin-1). These observations suggest that the use of HCQ (or targeting specific autophagic mediators) may be detrimental to both human endometrial and endometriotic cell survival.

To determine whether treatment with HCQ alters the formation of endometriotic lesions, we used an induced model of murine endometriosis in which mice receive injections of uterine horn fragments that develop into lesions within 2 weeks.^19, 20^ We treated recipient (endometriosis-induced) mice with 60 mg/kg HCQ^21^ or phosphate-buffered saline (PBS; Figure 1d). This treatment was repeated once every 7 days post-induction. Mice that were neither injected with uterine horn fragments nor treated were used as controls (Supplementary Figure 2a). All of the mice were killed at the same time (14 days after endometriosis induction for both the PBS and HCQ treatment groups). Ectopic lesions that developed in the recipient mice (white arrow; Supplementary Figure 2b) were counted, measured, and collected for RNA and protein analysis, as well as for histological staining. No lesions were observed in the control group (labeled as N; Supplementary Figure 2b). The majority (87.5%) of endometriosis-induced mice developed lesions. At the time of collection, we noted that the endometriotic lesions varied in size, color, and location across the treatment groups. As shown in Figure 1e, there was a significant reduction in the number of lesions that developed in mice treated with HCQ compared with those treated with PBS (*P*=0.0007; PBS-treated mice, *n*=24 (with a total of 46 lesions) and HCQ-treated mice, *n*=25 (with a total of 18 lesions)). However, there was no significant difference in lesion size or volume between these two treatment groups (Figure 1e).

A randomly selected subset of the collected lesions and uterine horns were processed for staining with hematoxylin and eosin (H&E) (pathologically confirmed endometriotic lesions from PBS- and HCQ-treated mice, *n*=15 each; uterine horns derived from PBS-treated mice, *n*=10; and uterine horns derived from HCQ-treated mice, *n*=10). Interestingly, as shown in Figure 1f, we observed an irregular epithelium pattern in 5 out of 10 uterine horns derived from HCQ-treated mice compared with those derived from PBS-treated mice. In addition, we noted that the ectopic growths from HCQ-treated mice did not histologically resemble endometriotic lesions (i.e., did not contain the expected glandular components), whereas those treated with PBS did (Figure 1f, black arrowheads indicate glandular compartments, while black arrows indicate epithelial cells within the lesions) (*P*=0.03, per Fisher's exact test). Taken together, these results reveal that HCQ reduces the number of endometriotic lesions and alters the cellular organization within these tissues.

### Altered levels of peritoneal macrophages and IP-10 cytokine from HCQ-treated mice

**Figure 2:**
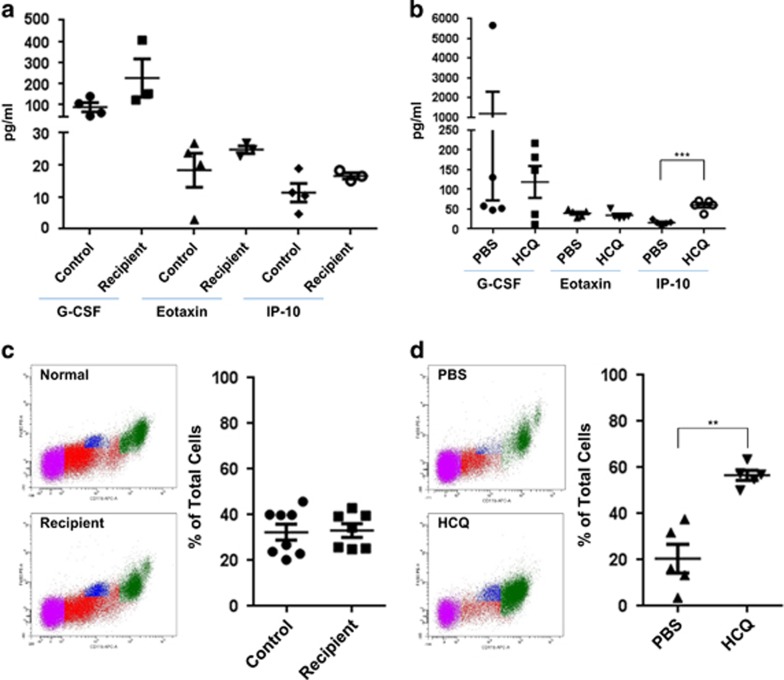
HCQ treatment increases the numbers of peritoneal macrophages and chemokine levels of IP-10. (**a**) Peritoneal fluid collected for control and recipient mice were analyzed for 32 cytokines/chemokines. The data are presented as a dot plot (showing individual sample values), and the line indicates the average±S.E.M. (**b**) Peritoneal fluid was collected from HCQ- and PBS-treated mice for cytokine/chemokine analysis. The data are presented as a dot plot (showing individual sample values) and the line represents average±S.E.M. (**c**) Macrophages were stained with CD11b and F4/80 antibodies and then analyzed by flow cytometry. Representative images of the raw flow cytometry data are shown. The data are presented as a dot plot (showing individual sample values) and the line represents average±S.E.M. (**d**) Macrophages were collected from the same specimens analyzed in (**b**). Macrophages were stained as described in (**c**). Representative images of the raw flow cytometry data are shown. The data are presented as a dot plot (showing individual sample values) and the line represents average±S.E.M

To investigate changes in the inflammatory response to endometriosis, we quantified 32 cytokines/chemokines in the peritoneal fluid collected from control (*n*=4) and recipient (*n*=3) mice using a mouse cytokine and chemokine magnetic bead panel assay. Of the 32 analyzed cytokines/chemokines, we identified that G-CSF, eotaxin, and IP-10 (10 kDa interferon-*γ*-induced protein; also known as CXCL10) were within the sensitivity and detection limits of the assay; however, there were no significant differences in these proteins between control and recipient mice (Figure 2a). In contrast, we identified that IP-10 was significantly increased (*P*=0.0079) in peritoneal fluid obtained from HCQ-treated mice (*n*=5) compared with PBS-treated mice (*n*=5), whereas G-CSF and eotaxin remained unchanged (Figure 2b).

As previous research identified a significant increase in macrophage numbers in endometriosis-induced mice^22^ and that HCQ can inhibit cytokine production in human macrophages,^23^ we therefore assessed macrophage numbers in control, recipient (untreated), PBS-treated, and HCQ-treated recipient mice. There was no significant change in the macrophage numbers present in the peritoneal cavity of control and endometriosis-induced mice at the time of sample collection (2 weeks post-induction), using the canonical macrophage markers CD11b and F4/80 (Figure 2c). However, we did find a significant increase (*P*=0.0079) in macrophage numbers in HCQ-treated mice compared with PBS-treated mice (Figure 2d). These data indicate that HCQ alters the inflammatory response of endometriosis-induced mice.

### HCQ induces cellular disorganization in murine endometriotic lesions and eutopic endometrial

**Figure 3:**
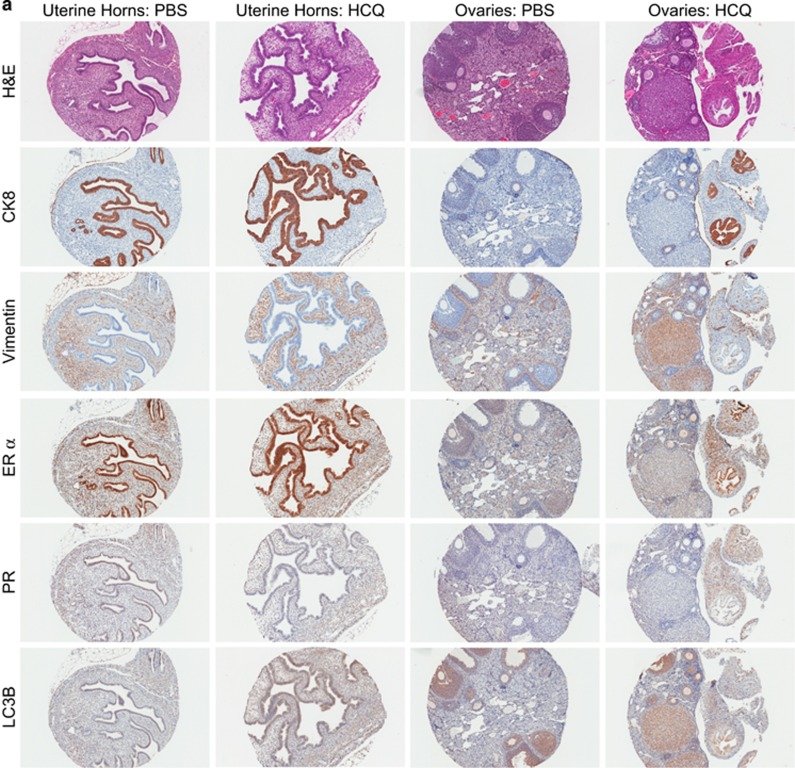

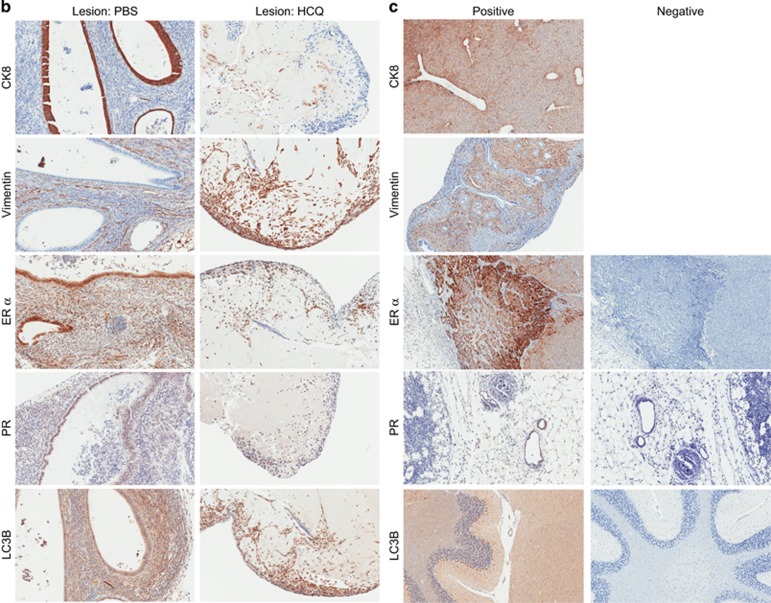
Immunohistochemical analyses of murine endometria, ovaries, and lesions. (**a**) Representative immunohistochemical images of uterine horns and ovaries from PBS- and HCQ-treated mice are shown. The cores were processed for H&E staining as well as epithelial and stromal markers (CK8 and vimentin, respectively), ovarian hormone receptors (ER *α* and PR), and the autophagy marker LC3B. (**b**) Representative immunohistochemical images are shown from lesions collected from PBS- and HCQ-treated mice. The sections were stained as described in (**a**). (**c**) Representative images of antibody immunohistochemical staining controls (both positive and negative staining) are shown. For LC3B, mouse brain was used as a positive staining control tissue. For PR and ER *α*, mouse mammary glands were used as positive staining control tissues. For vimentin and CK8, mouse uterine horns were used as positive staining control tissues. Negative staining controls were performed in the absence of primary antibody. The images of PR-positive and -negative staining controls are shown at 20 × magnification; all other images are shown at 10 × magnification

To determine whether HCQ treatment alters the histopathology (tissue organization) of the recipient's uterine horns and other tissues, we developed a murine tissue microarray (TMA) comprised of 113 cores and performed H&E as well as immunohistochemical staining. The TMA contained uterine horns and ovaries from 10 PBS- and 10 HCQ-treated mice, as well as a mammary gland, a kidney, a lymph node, and a small intestine from a PBS-treated mouse for use as antibody controls. Based on H&E staining, we observed that the luminal epithelium of the uterine horn endometrium from HCQ-treated mice had an irregular pattern (Figure 3a). However, vimentin and cytokeratin 8 (CK8) appeared to be appropriately localized to the stromal and epithelial compartments, respectively, independently of HCQ treatment. As expected, estrogen receptor *α* (ER *α*) was primarily localized to the epithelial cell layer of the endometrial glands, whereas progesterone receptor (PR) appeared to be evenly distributed between the stromal and epithelial cell compartments;^24^ however, the PR staining was comparatively much weaker to that for ER *α*. Again, no differences were noted in the tissues from PBS- and HCQ-treated mice for ER *α* and PR staining pattern or intensity. LC3B expression appeared more intense in HCQ-treated mice relative to PBS-treated mice in both the stromal and epithelial compartments (Figure 3a). We also stained for the same immunohistochemical markers in ovaries, but we did not observe any marked differences in the intensity or localization pattern of any of these proteins in these tissues from HCQ-treated mice relative to those from PBS-treated mice.

A certified pathologist confirmed epithelial and stromal components in the lesions analyzed. Lesions (independent blocks and not on the above-described TMA (see Materials and Methods)) were also immunostained for CK8, vimentin, ER *α*, and PR, and LC3B (Figure 3b). The epithelial cells of the glands were positive for CK8, ER *α*, and PR expression, which provides supporting data that the collected lesions originated from endometrial tissue (Figure 3b). Interestingly, there was an absence of glandular components in four out of the seven stained lesions from HCQ-treated mice as demonstrated by CK8, ER *α*, and PR immunohistochemical staining. These results (with the H&E data presented in Figure 1f) suggest that HCQ alters the organization of ectopic growths in the murine model of endometriosis. Figure 3c displays representative images of positive and negative staining controls for the antibodies used.

### Induction of endometriosis downregulates mRNA and protein expression of autophagic markers in ectopic compared with eutopic murine endometrium

**Figure 4:**
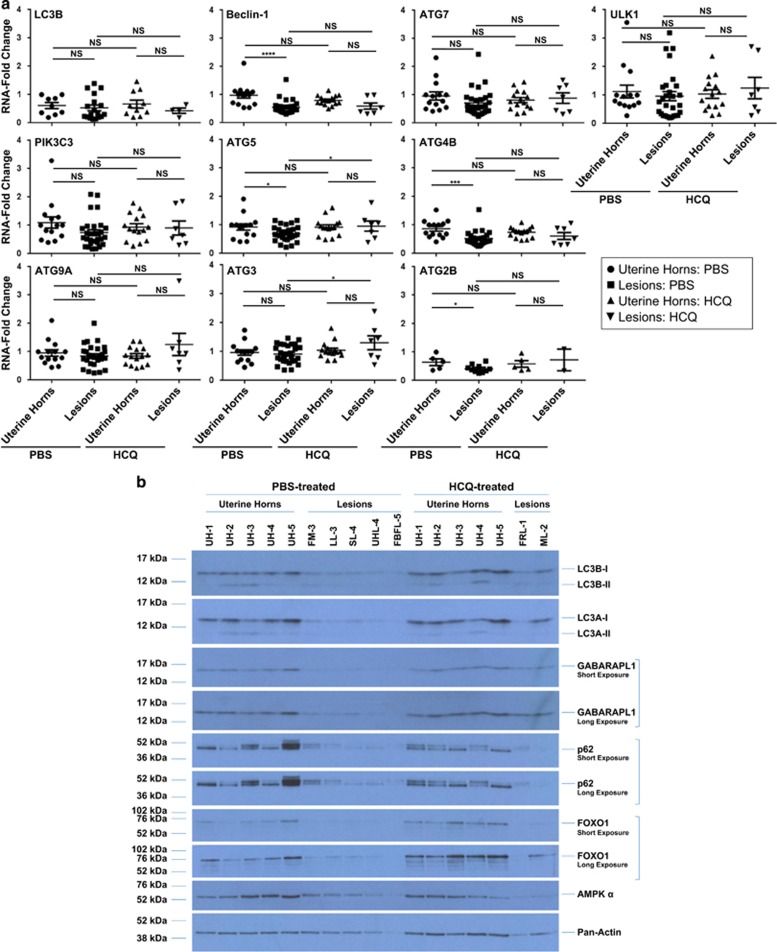

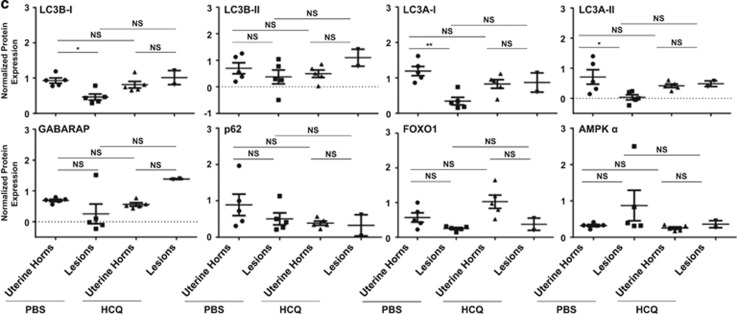
Autophagy gene expression and protein levels are decreased in uterine horns and lesions from endometriosis-induced mice, independently of HCQ treatment. (**a**) A subset of samples was analyzed to quantify transcript levels of autophagic markers by real-time PCR. The line indicates average±S.E.M. (**b**) Protein expression was assessed by western blot analyses across the indicated groups using the indicated antibodies. Pan-actin was used as a loading control. The selected western blot presented in the panel is representative of the data obtained across all of these specimens analyzed and includes: (1) PBS-treated mice: uterine horns (*n*=5); (2) HCQ-treated mice: uterine horns (*n*=5); (3) PBS-treated mice: lesions (*n*=5); and (4) HCQ-treated mice: lesions (*n*=2). UH, uterine horns; FM, mass located near fat; LL, lesion located on the liver; SL, lesion located near the surface of the peritoneal cavity; UHL, lesion located near the uterine horn; FBFL, blood-filled lesion located near the fat; FRL, red lesion located near the fat; ML, lesion located near the mesentery. (**c**) Densitometric analyses of the presented western blots (as presented in (**b**)) are shown (presented as average±S.E.M.). For GABARAPL1 and p62, analysis of the short exposure is shown. For FOXO1, analysis of the long exposure is shown

To determine whether the expression of autophagic mediators in uterine horns and lesions differs between PBS- and HCQ-treated mice, we used real-time PCR to quantify the mRNA transcript levels of 10 major molecules involved in the autophagic pathway. In PBS-treated animals, we determined that the mRNA levels of ATG5 (*P*=0.0294), ATG4B (*P*=0.0004), ATG2B (*P*=0.0440), and beclin-1 (*P*<0.0001) were significantly decreased in the analyzed endometriotic lesions compared with uterine horns (uterine horns from PBS-treated mice, *n*=14; lesions from PBS-treated mice, *n*=28; uterine horns from HCQ-treated mice, *n*=15; and lesions from HCQ-treated mice, *n*=7) (Figure 4a). Owing to limited sample availability, LC3B and ATG2B were analyzed using a smaller number of samples (i.e., for LC3B: uterine horns from PBS-treated mice, *n*=9; lesions from PBS-treated mice, *n*=18; uterine horns from HCQ-treated mice, *n*=10; and lesions from HCQ-treated mice, *n*=4; For ATG2B: uterine horns from PBS-treated mice, *n*=5; lesions from PBS-treated mice, *n*=10; uterine horns from HCQ-treated mice, *n*=5; and lesions from HCQ-treated mice, *n*=2). No significant differences were noted between lesions and uterine horns from HCQ-treated C57BL/6 mice (likely due to smaller lesion numbers available in the HCQ group, although similar trends were apparent). However, lesions obtained from these HCQ-treated mice had a significant increase in ATG5 (*P*=0.0499) and ATG3 (*P*=0.0248) compared with lesions from PBS-treated mice (Figure 4a).

We also used the Balb/c mouse strain for the induction model to demonstrate that the changes observed in autophagy gene expression are independent of the mouse genetic strain used.^20^ In this model, we identified that the mRNA levels of ATG7 (*P*=0.0174), ATG4B (*P*=0.0020), and beclin-1 (*P*<0.0001) were significantly reduced in endometriotic lesions (*n*=8) compared with uterine horns (*n*=8) derived from the same recipient mice (Supplementary Figure 3).

We next analyzed the protein levels of autophagic markers in both lesions and uterine horns from PBS- and HCQ-treated mice (Figure 4b) from the following groups: (1) PBS-treated mice: uterine horns (*n*=15); (2) HCQ-treated mice: uterine horns (*n*=15); (3) PBS-treated mice: lesions (*n*=10); and (4) HCQ-treated mice: lesions (*n*=7). LC3B-I, LC3B-II, LC3A-I, and LC3A-II were decreased in lesions compared with uterine horns from both PBS- and HCQ-treated mice. Expression of GABARAPL1-I (GABA(A) receptor-associated protein like 1) was detected in uterine horns collected from both groups of treated mice and was decreased in the lesions; however, the conjugated form, GABARAPL1-II, was not observed in any of the murine specimens. We also observed a decrease in p62 in endometriotic lesions relative to uterine horns that was independent of HCQ treatment. FOXO1 and AMPK*α* protein levels in the uterine horns were variable among the samples analyzed, although they were both reduced within the lesions (Figures 4b and c). To determine whether HCQ treatment altered the expression of autophagic mediators in other organs, we harvested various tissue specimens (kidneys, thymus, spleen, lung, pancreas, heart, and liver) from each treatment group (five PBS-treated mice and five HCQ-treated mice) and assessed LC3B levels (Supplementary Figure 4). Among these tissues, only the lung and heart showed differences in LC3B-II expression following HCQ treatment. Overall, these results suggest that the protein expression of autophagic mediators is dysregulated in endometriotic lesions and is not affected by treatment with HCQ in the majority of tissues analyzed, including uterine horns.

### RNA expression of autophagic markers is dysregulated in eutopic endometria upon induction of endometriosis

**Figure 5:**
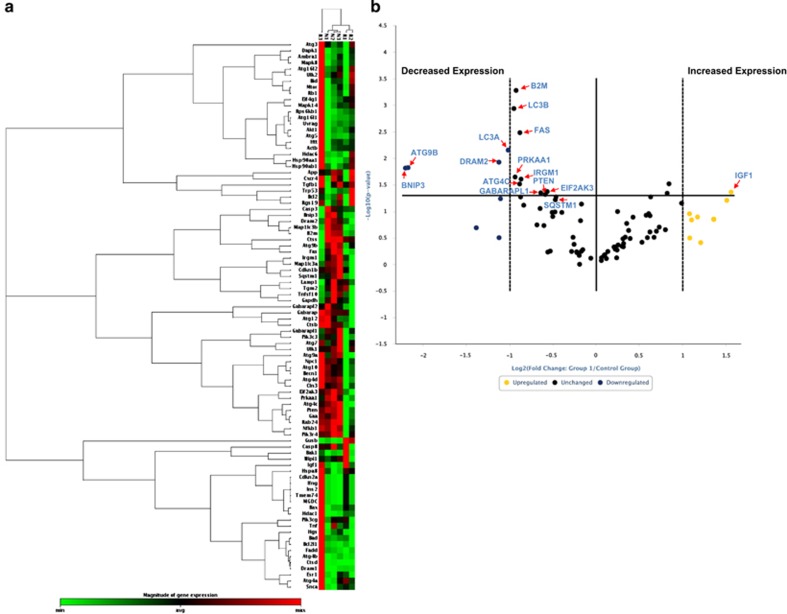

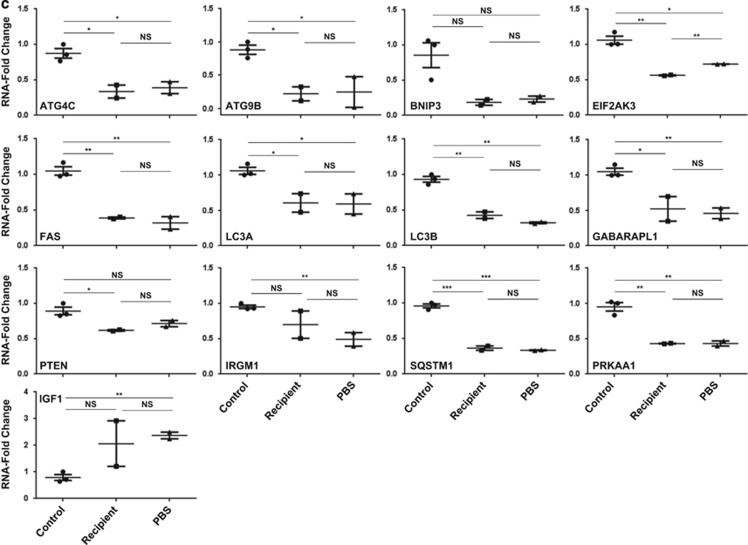
Decreased RNA expression of autophagic markers in eutopic endometria from mice with endometriosis relative to controls. (**a**) The selected RNA samples from non-induced (control) and endometriosis-induced mice were analyzed using an RT^2^-PCR profiler array specific for 84 autophagy-related genes. A heat map shown depicting the measured *C*_T_ values across these specimens is presented. (**b**) A volcano plot is shown from the analyzed data presented in (**a**). The horizontal axis indicates significance (*P*=0.05) if targets are above the line; the dotted vertical bars denote at least a two-fold change in gene expression if targets are ⩾−1 or 1 Log_2_ units. Red arrows indicate the autophagic markers that were decreased >2-fold with a *P*-value of <0.05. (**c**) Validation using TaqMan real-time PCR of specimens used in (**a**)

Evidence is accumulating that the eutopic endometria from patients with endometriosis differs markedly from the eutopic endometria from endometriosis-free subjects.^25, 26^ To identify changes in the expression of key autophagic markers in this context, we used an RT^2^-PCR autophagy focused profiler array to analyze RNA isolated from uterine horns from control (non-induced) and recipient (untreated). In addition, we compared the uterine horns from recipient mice with those from PBS-treated recipient mice to verify that there was no significant change that occurred upon intraperitoneal injection with PBS. Three representative samples were selected from each group based on RNA quality. A heat map comparing gene expression in RNA isolated from uterine horns from control mice to recipient mice is shown in Figure 5a; the results indicate that there is a subset of autophagy genes that is differentially expressed. A volcano plot is shown in Figure 5b that displays the fold changes in autophagy genes in eutopic endometria between recipient and control mice. We identified 13 dysregulated genes (with statistical significance) between these two groups of samples. Insulin-like growth factor 1 (IGF1) was the only autophagic marker that was significantly increased (*P*=0.044); the remaining 12 markers were all significantly decreased (BNIP3 (BCL2/adenovirus E1B 19 kDa interacting protein), *P*=0.015; ATG9B, *P*=0.015; LC3A, *P*=0.007; LC3B, *P*=0.0012; protein kinase AMP-activated, α1 catalytic subunit (PRKAA1), *P*=0.023; ATG4C, *P*=0.031; FAS, *P*=0.003; IRGM1 (immunity-related GTPase family M1), *P*=0.025; GABARAPL1, *P*=0.045; PTEN (phosphatase and tensin homolog), *P*=0.048; EIF2AK3 (eukaryotic translation initiation factor 2-*α* kinase 3), *P*=0.043; and SQSTM1 (sequestosome 1), *P*=0.054). As shown in Supplementary Figure 5, we did not observe any significant changes upon PBS treatment in the RT^2^-PCR array.

To validate these ‘top hits' (i.e., increased by at least two-fold with *P*<0.05) identified from the autophagic pathway RT^2^-PCR profiler array, we performed real-time PCR using TaqMan FAM-labeled probes/primers (Figure 5c and Supplementary Table 1). Using this approach, we validated 10 of the 13 ‘top hits' (Figure 5c): ATG4C (*P*=0.0167), ATG9B (0.0113), EIF2AK3 (*P*=0.0068), FAS (*P*=0.0034), LC3A (*P*=0.0306), LC3B (*P*=0.0040), GABARAPL1 (*P*=0.0360), PTEN (*P*=0.0295), SQSTM1 (*P*=0.0008), and PRKAA1 (*P*=0.0065) were significantly reduced. Although the majority of the tested autophagic markers were not significantly changed upon PBS treatment relative to recipient (untreated), we did identify that the expression of EIF2AK3 (*P*=0.0014) was increased (Figure 5c). Taken together, these data suggest that autophagy is dysregulated in the eutopic endometria of endometriosis-induced mice.

### Increased LC3 protein and lipid droplets in eutopic endometria of endometriosis-induced mice compared with eutopic endometria of controls

**Figure 6:**
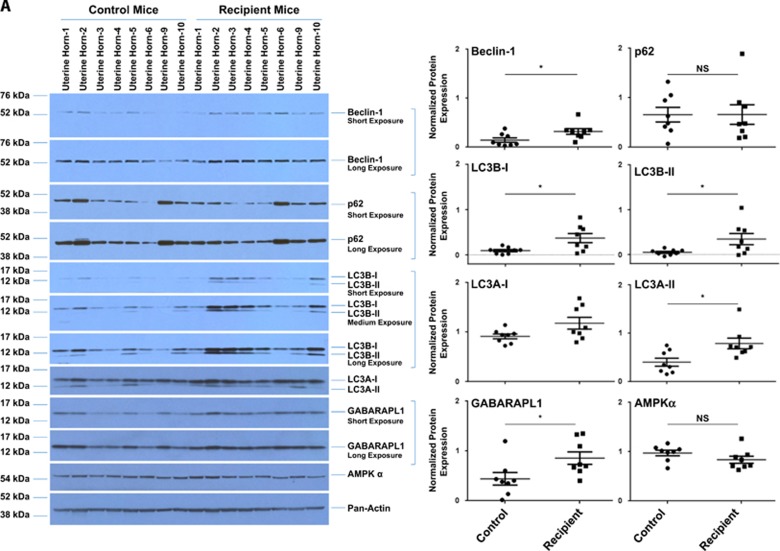

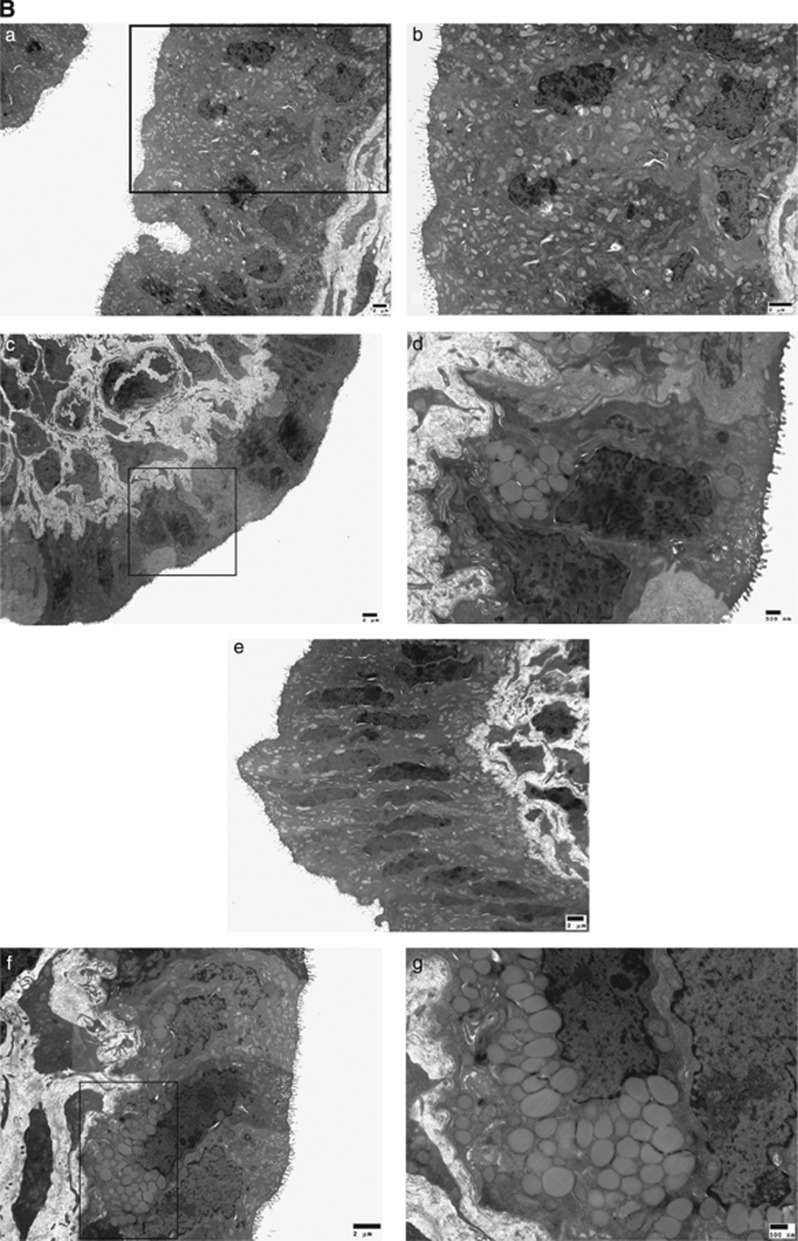
Increased protein expression of autophagy markers in the eutopic endometria from endometriosis-induced mice relative to controls. (**A**) Tissue homogenates from uterine horns from non-induced and endometriosis-induced mice were analyzed for protein expression by western blotting (left panels). Pan-actin was used as the loading control. A representation for 8 out of 10 total samples for both control and recipient groups is shown. Densitometric analyses of the presented western blots (right panels) are shown (presented as average±S.E.M.). (**B**) Uterine horns from control and endometriosis-induced mice were analyzed by TEM and representative images of epithelial cells are shown. (a–d) Eutopic endometria from control mice; (e–g) eutopic endometria from endometriosis-induced mice. The images on the right are magnifications of the indicated boxed region in the respective left image

To determine whether the RNA level changes of key autophagic markers observed between the eutopic endometria of endometriosis-induced mice (*n*=10) and non-induced (control) mice (*n*=10) translated to protein level changes, we assessed their protein levels via western blot analyses. As shown in Figure 6A and Supplementary Table 2, beclin-1 (2.20-fold change, *P*=0.0330), LC3B-I (4.00-fold change, *P*=0.0185), LC3B-II (6.76-fold change, *P*=0.0364), LC3A-II (1.97-fold change, *P*=0.0135), and GABARAPL1 (1.95-fold change, *P*=0.0334) were significantly increased in uterine horns from endometriosis-induced mice relative to those from control mice. LC3A-I and LC3B-I have an expected molecular weight of ~16 kDa, whereas LC3A-II and LC3B-II have an expected molecular weight of ~14 kDa.^27^ When we assessed GABARAPL1 expression, we did not detect the conjugated form, suggesting that the primary form expressed in these tissues is the cytosolic form (GABARAPL1-I). To assess if the increased levels of LC3B were specific to the uterine horns in the endometriosis-induced mice, we analyzed LC3B protein levels in homogenates prepared from kidneys, thymus, spleen, lung, pancreas, heart, liver, and ovaries from both recipient (*n*=3) and control (*n*=4) mice. Out of the nine tissues analyzed, only the left kidney, spleen, and liver appeared to show differences in LC3B-II levels (Supplementary Figure 6).

To test whether the observed increases in LC3A and LC3B correlated with an increase in autophagosome formation in the eutopic endometria of endometriosis-induced mice, we performed transmission electron microscope (TEM) (Figure 6B). Although no autophagosomes were identified in eutopic endometria from control mice (Figure 6Ba–d) and eutopic endometria from endometriosis-induced mice (Figure 6Ce–g), we observed an increase in lipid droplet numbers in the epithelial cells of eutopic endometria from endometriosis-induced mice. In addition, we also observed more ‘unhealthy' electron-dense epithelial cells in uterine horns from endometriosis-induced mice (Figure 6Ce) compared with control mice (Figure 6Ba). Taken together, these results suggest that expression of autophagic mediators (i.e., LC3) is dysregulated in the eutopic endometria of endometriosis-induced mice, which is associated with an accumulation of lipid droplets in the epithelial cells.

### Immunohistochemical staining of LC3B in the epithelium and stromal components of eutopic and ectopic endometrium in patients with endometriosis

**Figure 7:**
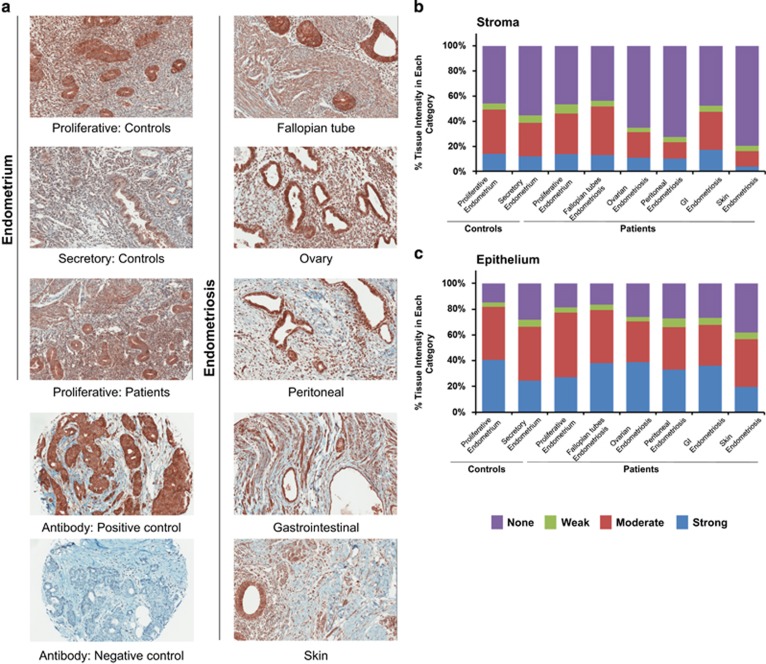
LC3B is predominantly localized in the epithelium of ectopic and eutopic endometrium. (**a**) LC3B immunostaining was performed on a TMA containing biopsy cores: eutopic endometrium (from controls and endometriosis patients) and endometriotic lesions (from ovaries, fallopian tubes, peritoneal, gastrointestinal, and skin). Mammary glands were used as a positive control tissue and the negative antibody staining control was performed in the absence of primary antibody. All images were captured at × 20 magnification. LC3B immunostaining was analyzed using the H-score system. The average immunostaining in the (**b**) stromal and (**c**) epithelial compartments were categorized as strong, moderate, weak, and no expression

We next addressed the cellular localization of LC3B within human eutopic and ectopic endometrium by applying an immunohistochemical approach using a human endometriosis and endometrium TMA.^28^ Representative immunohistochemical images for endometrium (controls and patients) and lesions (fallopian tubes, ovaries, peritoneal, gastrointestinal, and skin) are shown (Figure 7a). We noted that LC3B was localized primarily to the epithelium, although staining was also noted in the stroma. To quantify the intensity of LC3B expression at these specific cellular locations, we segmented the sections using the H-score system into strong, moderate, weak, or no expression (Figures 7b and c). The proportion of strong expression was elevated in the epithelial cells of the proliferative endometrium from cases (40.6%) and those from ovarian and fallopian tube lesions (38.8% and 38.0%, respectively). The endometriotic tissue with the highest proportion of strong stromal expression was the gastrointestinal tract (GI) (17.4%), followed by proliferative endometrium from controls (14.1%), proliferative endometrium from endometriosis patients (13.6%), and secretory endometrium from controls (12.0%) (Figures 7b and c). We noted a significant difference in LC3B expression in the epithelium of secretory endometrium compared with proliferative endometrium (*P*=0.0193) (Supplementary Figure 7, lower panel). We also found significantly increased expression in the epithelium of fallopian tube and ovarian endometriotic lesions compared with epithelium from the secretory endometrium of controls (*P*=0.0220 and *P*=0.0097, respectively). In the stroma of peritoneal endometriotic lesions, LC3B was decreased compared with the stroma of proliferative endometrium from controls (*P*=0.0101). In addition, relative to the stroma, positive LC3B immunostaining was significantly more elevated in the epithelial component of the lesions in the fallopian tube, ovarian, and peritoneum but not in lesions derived from the gastrointestinal tract and the skin (Supplementary Figure 7, upper panel). Thus, collectively, LC3B expression and localization was predominant in the epithelium relative to the stromal components in all tissue types assessed.

## Discussion

**Figure 8:**
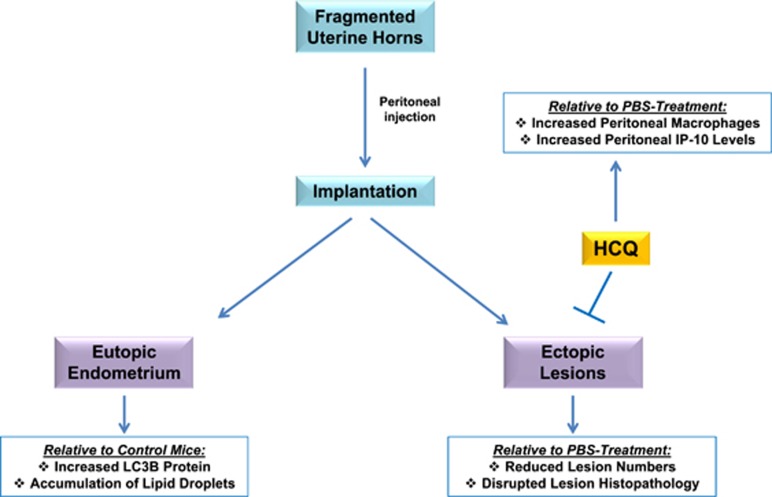
Schematic of overall results. Injected fragmented uterine horns implanted and developed in endometriotic lesions. LC3B and lipid droplets were elevated in recipient uterine horns compared with uterine horns from recipients, as indicated by protein analyses and TEM, respectively. HCQ reduced lesion numbers relative to PBS treatment. Moreover, IP-10 levels and macrophages increased in the peritoneal cavity of HCQ-treated mice compared with those treated with PBS

To our knowledge, our work presented herein is the first to investigate the effect of an autophagic flux inhibitor, HCQ, on endometriotic lesion development and histopathology. Our main findings are summarized in Figure 8. We found that HCQ treatment affected the survival of human endometrial and endometriotic cells *in vitro*, as well as decreased lesion numbers using an *in vivo* mouse model of endometriosis. The drug also appeared to have an effect on lesion histopathology (the absence of glandular components), but not on lesion size. We also identified that HCQ increases the number of macrophages and the IP-10 chemokine within the peritoneal cavity of a mouse model for endometriosis. Furthermore, we have identified that autophagic markers are differentially expressed in uterine horns from endometriosis-induced mice compared with those from control mice. Although we noted that LC3B protein level was increased in eutopic endometria of endometriosis-induced mice (compared with controls), we did not identify increased autophagosomes by TEM in these tissues. However, TEM showed that endometria from experimental mice are less healthy and contained an increased number of lipid droplets compared with endometria from control mice. Finally, we noted that LC3B was expressed and localized predominantly to the epithelia in all tissue types (relative to the stroma) of human endometrium and endometriotic lesions from diverse locations using a TMA approach.

Chloroquine, and derivatives of this agent (including HCQ), have been used to treat malaria, as well as inflammatory and autoimmune diseases.^29^ Endometriosis shares some characteristics with autoimmune disorders, such as increased inflammation, altered tissue-remodeling components, dysregulated cell death pathways, increased local and systemic cytokine levels.^30^ Inflammatory changes in the peritoneal cavity may be associated with lesion development.^31^ Other co-morbid autoimmune disorders (i.e., systemic lupus erythematosus, rheumatoid arthritis, multiple sclerosis) can coincide with the presence of endometriosis.^32, 33^ These autoimmune disorders can be treated with HCQ, which can antagonize communication among cells in the immune system that are inappropriately activated.^29, 34^ Therefore, HCQ could potentially be used as an effective therapeutic agent for other autoimmune related disorders, such as endometriosis. As described earlier, HCQ is considered a lysosomotropic agent where it increases the pH of acidic compartments and also inhibits the fusion of the autophagosome with the lysosome.^15, 16, 17^ We identified a marked reduction in cell survival with this agent in human endometriotic and T-HESC cells. The involvement of lysosomes in endometriosis has yet to be investigated in detail; to our knowledge, only one study describes changes in numbers of lysosomes in the endometrium of women with endometriosis.^35^ We also successfully knocked down ATG7 (in addition to other autophagic mediators) in T-HESC; we noted a marked increase in p21 protein expression, which was associated with attenuated cell viability with ATG7 siRNA (Supplementary Figure 1e); changes were observed with LC3B siRNA but only a subtle effect was noted with beclin-1 siRNA. However, with the exception of ATG5 and LC3B knockdown, we failed to detect the expected reduction in LC3-II to LC3-I ratio with PIK3C3 or beclin-1 siRNA (implicating a noncanonical autophagic pathway), as well as with ATG7 siRNA (implicating autophagy independent role). In support, it has been previously reported that ATG7 can lead to increased p21 via a DNA damage pathway,^36^ which appears to be independent of its canonical role in the autophagic pathway. Therefore, it is possible that the reduction in cell viability could have occurred via a similar noncanonical autophagy mechanism or independently of autophagy. Further work is necessary to clarify the nature of the contribution of these autophagic mediators to endometriotic cell survival and lesion development.

Although we identified increases in ATG5 and ATG3 in lesions from HCQ-treated mice relative to PBS-treated mice, the majority of the remaining autophagic markers that we studied were unchanged; however, we recognize that this may be because of a limited lesion number sample size obtained from HCQ-treated mice. As we observed fewer glandular structures and epithelial cells in lesions from endometriosis-induced mice treated with HCQ, it is possible that this drug is affecting the epithelial cells of endometriotic lesions more than the stromal cells, thus leading to a decrease in lesion number and quality. Our analyses of autophagic markers (by real-time PCR or western blotting) did not distinguish between stromal and epithelial cells; therefore, further tests would be required to dissect the response between the two cell types that may be leading to the observed decrease in lesion number.

HCQ treatment increased the levels of IP-10 and the numbers of macrophages in the peritoneal cavity in our mouse model of endometriosis. In humans, IP-10 has been reported to attract monocytes and T lymphocytes, as well as decrease angiogenesis.^37, 38^ A decrease in IP-10 peritoneal levels has been reported in advanced stages of endometriosis in patients, and thus decreased levels of IP-10 may contribute to lesion development, permitting angiogenesis, and decreasing recruitment of natural killer cells and their cytolytic effects.^39^ Therefore, an increase in peritoneal IP-10 levels in HCQ-treated mice may have created an unfavorable environment for lesion development. Whether IP-10 modulates the autophagic pathway requires further investigation. It is interesting that another chemokine CXCL12 has recently been implicated in modulating autophagy in human secretory phase endometrial stromal cells.^40^ Additional experiments will be required to determine whether the HCQ effects leading to the increased IP-10 levels corresponds with the observed decrease in lesion number and the mechanisms whereby IP-10 could be exerting its anti-endometriotic growth effects.

A previous study reported altered gene expression in eutopic endometria from patients with endometriosis compared with those of controls,^41^ suggesting that endometriosis may be altering the endometria of patients. In our study, we noted that the mRNA expression of autophagic markers was generally decreased in eutopic endometria from endometriosis-induced mice when compared with eutopic endometria of control mice. Specifically, we found that transcript levels of beclin-1, ATG5, ATG4B, and ATG2B were significantly decreased in endometriotic lesions compared with uterine horns (from endometriosis-induced mice treated with PBS). Beclin-1 is required for vesicle nucleation during autophagosome formation and forms a complex with UVRAG to regulate PIK3C3.^10^ ATG5 and ATG4B are required to generate the cytosolic form of LC3, LC3-I.^42, 43, 44^ In addition, we found that LC3B protein levels were decreased in lesions compared with uterine horns. Taken together, these changes suggest that dysregulation of autophagic markers may alter the autophagic response in endometriosis. Further research is needed to determine whether autophagosome numbers are altered within ectopic lesions. In contrast to the observations in the lesions, we identified increased protein levels of beclin-1, LC3B, LC3A, and GABARAPL1 in uterine horns from endometriosis-induced mice compared with uterine horns from controls, with a concurrent increase in lipid droplets. Lipid droplets have been previously described in bovine endometrial epithelial cells,^45^ and recent findings have implicated LC3 in intracellular lipid droplet formation.^46^ It is possible that the observed increases in LC3B protein are associated with the accumulated lipid droplets, but further work is needed to understand the clinical implications of this finding.

The observed changes in the expression of autophagic markers in eutopic endometria from induced mice could be explained by an activated local immune/inflammatory response caused by transferring endometrial fragments into the peritoneal cavity.^3^ Although we did not observe an increase in inflammatory markers or macrophage levels 2 weeks post-induction, we propose that any inflammatory response that occurred may have been diminished by the time of sample collection. Taken together, these observations may reveal why endometriosis is able to recur in women after surgical removal of endometriotic lesions.^47^ Whether the eutopic endometrium of women with endometriosis is altered before lesion establishment or whether it becomes more compromised after the initiation and establishment of the disease remains unclear and requires further study.

In contrast to the reduced expression of LC3B protein in lesions obtained from the mouse model of endometriosis (relative to uterine horns isolated from the same diseased mice), we identified an increase in LC3B levels in epithelial cells of endometriotic lesions and in eutopic endometria of endometriosis patients compared with endometrium from controls using immunohistochemistry. We propose that this difference may be due to the possibility that the murine endometriosis lesions are equivalent to an early stage of endometriosis in patients. As the human disease is not diagnosed until a delay period of 7–10 years,^48, 49^ the nature of the isolated lesions from human may be more advanced. In addition, the endometrium controls on the TMA are from women with other gynecological problems, which is a limitation of our study. Despite these limitations, the results obtained with the human TMA are more close to the real-life scenario of patient heterogeneity in clinical presentations and history of disease (previous treatments, years with disease) and still the changes in LC3B remained significant.

Whether the aforementioned observed expression changes implicate autophagy dysregulation in a pro-survival or pro-death manner is currently unknown with regard to endometriotic lesion establishment. Additional studies will be required to determine how autophagy is regulated to allow development of endometriosis. Our results also show potential for a novel therapeutic approach for treating patients with this disease. Patients with autoimmune diseases (i.e., lupus and arthritis) appear to tolerate HCQ treatment well; side effects include retinal damage with only rare reports of systemic reactions.^50^ Thus far, there seems to be no adverse effects of this drug on fertility,^51^ which makes HCQ superior to the current use of estroprogestinic treatments.

## Materials and Methods

### Ethics and TMA

All protocols in this study were approved by the Institutional Review Board at the Ponce Research Institute (Ponce, Puerto Rico). Samples in the TMA were obtained in a de-identified manner from archived samples at a private pathology laboratory (Southern Pathology Laboratories in Ponce, Puerto Rico). Details regarding the human TMA used in this study have been described previously.^28^ Briefly, the TMA contains 164 cores, which is comprised of lesions (from the ovaries (*n*=29), fallopian tubes (*n*=16), peritoneum (*n*=34), skin (*n*=4), and gastrointestinal tract (*n*=7)), eutopic endometrium from endometriosis patients (*n*=22), as well as secretory (*n*=38) and proliferative (*n*=14) endometrium from endometriosis-free patients. The patients and controls recruited into this biobank were not currently or have been for at least 3 months before surgery on any hormonal medication.

### Animal handling

#### C57BL/6 mouse model

Five-week-old C57BL/6 female mice were purchased from Jackson Laboratories (Bar Harbor, ME, USA). All animals were maintained under standard 12-h photoperiod; food and water were available *ad libitum* throughout the study. All experimental procedures and animal care were approved by the Animal Care and Use Committee (IACUC) of the University of South Florida (R IS00000101), in accordance with the principles described in the Guide for the Care and Use of Laboratory Animals of the National Institutes of Health. All surgical procedures were performed under aseptic conditions using anesthesia. The mouse model of endometriosis was performed as described previously.^19, 20^ Donor animals received a peritoneal injection of 3 *μ*g per mouse of *β*-estradiol-17-valerate (Sigma, St. Louis, MO, USA); the dose used was based on previously reported data.^20^ One week after estrogen injection, donor animals were killed and each uterine horn was collected and minced using a Kirkland Tissue Mincer (Kirkland Products, Portland, OR, USA) with sterile normal saline. The minced material was centrifuged at 1500 r.p.m. for 1 min. Endometriosis was induced by injecting the uterine horn fragments intraperitoneally into the recipient animal. Mice were then randomly divided into two groups: HCQ treatment animals were intraperitoneally injected with 100 *μ*l of 60 mg/kg of HCQ (No. AC26301; Fisher Scientific, Pittsburgh, PA, USA), whereas control treatment animals received an intraperitoneal injection of 100 *μ*l sterile PBS. The dose for HCQ used was based on previously published data and was comparable to doses used in treating patients with autoimmune diseases.^21^ A second HCQ treatment was administered 1 week after endometriosis induction, using the same dose. Two weeks after induction, mice were killed and tissues (including lesions) were snap frozen into liquid nitrogen. Lesions were measured using a caliper. Volume of the lesions was calculated according to the formula: 4/3π*r*^2^*R*.^52^

#### Balb/c mouse model

Eight-week-old Balb/c female mice were obtained from the Charles River Laboratories (Calco, Como, Italy) and handled as described previously^19, 20^ and in accordance with the European Union guidelines, as well as with the approval of the Institutional Animal Care and Use Committee of San Raffaele Scientific Institute (Protocol No. 484) (Milan, Italy). Briefly, donor mice were injected with 17*β*-estradiol (AMSA, Rome, Italy; 3 *μ*g per mouse) and killed 1 week later. The uterus was removed and fragmented, after scraping to remove the myometrium, using scissors. The endometrial tissues were weighed and resuspended in saline with ampicillin (1 mg/ml). Two recipient mice received an intraperitoneal injection, using a syringe containing half of the resuspension (day 0). Mice were killed by administering a lethal dose of anesthetic on day 12. The abdomen was opened and lesions were isolated and collected by an operator blinded to the experiment.

### Cell culture of life-extended human endometriotic and T-HESC cells, HCQ treatment, siRNA transfection, and survival assay

Primary human endometriotic cells culture conditions and life extension have been described previously.^18^ These cells were derived from peritoneal (‘C') and ovarian (‘D') lesions obtained from two independent patients. These were assessed separately as described below. Briefly, cells were maintained in MCDB 131:Medium 199 (1 : 1 ratio) supplemented with 8% fetal bovine serum (FBS), penicillin/streptomycin, and insulin/transferrin/selenium (ITS). Cells were life extended using simian virus 40 large T antigen. Retroviral particles generated in HEK293T were used to infect the primary cells. Media containing puromycin (2.5 *μ*g/ml) was used to select primary cells resistant colonies. In addition, we obtained the T-HESC cell line, which are human endometrial stromal cells derived from a uterine myoma (ATCC, Manassas, VA, USA). This cell line was maintained in phenol red-free DMEM/F12 (1 : 1) containing 8% charcoal-dextran-treated FBS, 500 ng/ml puromycin, 1% ITS+ Premix (BD Bioscience, San Jose, CA, USA), and 15 mM HEPES. The cell lines used in the present study were tested to be mycoplasma negative and short tandem repeat profiled (Genetica DNA Laboratories, Cincinnati, OH, USA). Endometriotic cells were seeded at 50 000 cells per well in a 24-well plate, whereas T-HESC cells were seeded at 250 000 cells per well in a 6-well plate. A 50 mM HCQ (no. AC26301; Fisher Scientific, Pittsburgh, PA, USA) stock was prepared in PBS (and 0.22 *μ*m filter sterilized); it was used at a final concentration of 25 *μ*M in complete media.^18, 53^ Cells were treated for 18 h with HCQ before protein harvest and western blotting analyses. For survival studies, cells were seeded at a density of 5 000 cells per well in a 96-well opaque plate and treated with 25 *μ*M HCQ during 5 days. Cell viability was then assessed using CellTiter-glo reagent (Promega, Madison, WI, USA).^18^

For siRNA transfection studies, T-HESC cells were seeded at 350 000 cells per well in a 6-well plate. After overnight adherence, cells were then transfected with either non-targeting control siRNA, ATG5, beclin-1, ATG7, PIK3C3, or LC3B siRNA according to previously described methods.^18, 54^ The day after the second round of siRNA transfection, cells were reseeded at 5 000 cells per well in opaque 96-well plates. Three days after reseeding, cell viability was assessed using CellTiter-glo reagent as described above.

### Immunohistochemistry of LC3B

Samples in the TMA were collected in a de-identified manner from archived samples in a Pathology Lab as described in Human Subjects above. Briefly, slides were deparaffinized and stained using the automated system Ventana Discovery XT (Ventana Medical Systems, Tucson, AZ, USA) with EZ Prep solution. The heat antigen retrieval method was performed at a pH of 8.0. The primary antibody, LC3 (AP1802a), that detects LC3B was obtained from Abgent (San Diego, CA, USA) and diluted at a ratio of 1 : 25 in Dako antibody diluent (Dako, Carpenteria, CA, USA), followed by a 32 min incubation at room temperature. Human breast cancer tissue was used as a positive control and the primary antibody was omitted for the negative control. Ventana OmniMap anti-rabbit secondary antibody and the Ventana Medical Systems (Tucson, AZ, USA) as the detection system were used. Hematoxylin was used as the counterstain.

The LC3-stained TMA was then scanned using the Aperio ScanScope XT (Aperio, Vista, CA, USA) with a × 200 magnification and a 0.8 numerical aperture objective lens via the Basler tri-linear array detection. Each core was then segmented using the TMA block software associated with the Spectrum program (version 10.2.5.2352), followed by manual segmentation into epithelial and stromal regions under the supervision of a pathologist. Image analysis was performed using an Aperio Positive Pixel Count v.9.0 algorithm with the following thresholds: Hue Value=0.1; Hue Width=0.5; Color Saturation Threshold=0.04; IWP(High)=220; IWP(Low)=IP(High)=175; IP(low)=ISP(High)=100; ISP(Low)=0 to segment positive staining of various intensities. The data were then compiled for each core in the separate epithelium and stromal regions, which was represented by percent positivity, and then directly correlated with protein expression.

### RNA isolation, real-time PCR, and RT^2^-PCR

Total RNA was isolated using the RNeasy Kit following the manufacturer's instructions (Qiagen, Valencia, CA, USA). RNA concentration and purity was determined using a 1000 NanoDrop (Thermo Scientific, Pittsburgh, PA, USA). Lesion mass varied by samples, and this was reflected in the RNA amounts obtained (range of mass: 0.9–25 mg).

Three RNA samples from uterine horns, having a 260/280 ratio >1.8 and a 260/230 ratio >1.7, were selected from recipient, donor, HCQ-treated, and PBS-treated animals (12 samples in total) for RT^2^-PCR analyses. Synthesis of cDNA was performed using 0.5 *μ*g of total RNA, after DNA elimination step using the RT^2^ First Strand Kit as per the manufacturer's instruction (Qiagen). After DNA elimination, the reaction mix was incubated at 42 °C for 15 min, followed by 95 °C for 5 min using a DNA Engine Peltier Thermal cycler (Bio-Rad, Hercules, CA, USA). A total of 102 *μ*l of the cDNA reaction mix was added to the master mix containing 1248 *μ*l of RNAse-free water and 1350 *μ*l of 2x RT^2^ SYBR green master mix. Twenty-five microliters of the master mix were carefully added to each well of the RT^2^ profiler PCR autophagy array. Quantification was performed using the Applied Biosystems cycler (Life Technologies, Grand Island, NY, USA). The PCR cycling program included activation for 10 min at 95 °C, followed by 40 cycles for 15 s at 95 °C with 1 min at 60 °C. The PCR cycling program finalized with a melt-curve analysis and data was analyzed using the Qiagen web-based software (http://www.SABiosciences.com/pcrarraydataanalysis.php).

For real-time PCR studies, the One-step Master Mix (Applied Biosystems, Foster City, CA, USA) was used with the following probes and primers as described previously:^18^ LC3B, Mm00782868_sH; ATG4B, Mm01701111_m1; ATG9A, Mm01264420_m1; ATG5, Mm00504340_m1; ATG7, Mm00512209_m1; ATG3, Mm00471287_m1; PIK3C3, Mm00619489_m1; ULK1 (unc-51-like autophagy-activating kinase 1), Mm00437238_m1; ATG9B, Mm01157883_g1; Beclin-1, Mm01265461_m1; ATG2B, Mm00512973_m1; ATG4C, Mm01259886_m1; BNIP3, Mm01275600_g1; EIF2AK3, Mm00438700_m1; FAS, Mm01204974_m1; LC3A, Mm00458725_g1; GABARAPL1, Mm00457880_m1; IGF1, Mm00439560_m1; IRGM1, Mm00492596_m1; SQSTM1 (p62), Mm00448091_m1; PRKAA1, Mm01296700_m1; PTEN, Mm00477208_m1. *C*_T_ values were normalized to *β*-actin (Mm00607939_s1) and RNA fold changes were determined using the 2^−ΔΔC^_T_ equation.

### Protein isolation, SDS-PAGE, and western blot analyses

Tissues used for protein analyses included uterine horns, ectopic lesions, ovaries, thymus, kidneys, heart, pancreas, spleen, and liver. Samples were flash frozen in liquid nitrogen and stored at −80 °C until use. Tissues were homogenized in ice-cold lysis buffer containing 1% Triton X-100, 50 mM HEPES, 150 mM NaCl, 1 mM MgCl_2_, 1 mM EGTA, 10% glycerol, and protease inhibitor cocktail (Roche, Indianapolis, IN, USA) using a PowerGen 125 homogenizer (Fisher Scientific, Pittsburgh, PA, USA). Samples were centrifuged at 14 000 r.p.m. for 10 min at 4 °C. The supernatants were collected and total protein concentration was determined using the BCA assay (Thermo Scientific, Rockford, IL, USA), and a BioTek synergy 2 microplate reader (BioTek, Winooski, VT, USA). Samples were normalized and then run onto 10 or 12% SDS-polyacrylamide gels prepared in a Criterion Cassette system (Bio-Rad, Hercules, CA, USA) as described previously.^54^ The following antibodies and dilutions were used: LC3B rabbit polyclonal (no. 2775, 1 : 1 000), LC3A rabbit monoclonal (no. 4599 (D50G8), 1 : 1 000), Beclin-1 rabbit polyclonal (no. 3738, 1 : 1,000), GABARAPL1 rabbit monoclonal (no. 13733 (E1J4E), 1 : 1000), AMPK*α* rabbit monoclonal (no. 2603 (23A3), 1 : 500), FOXO1 rabbit monoclonal (no. 2880 (C29H4), 1 : 1000), and pan-actin rabbit polyclonal (no. 4968, 1 : 500) were all obtained from Cell Signaling Technology (Danvers, MA, USA). The p62 mouse monoclonal antibody (no. 610832, 1 : 1 000) was obtained from BD Biosciences (San Jose, CA, USA).

### Hematoxylin/eosin staining, TMA construction, and immunohistochemistry

Collected samples were immediately preserved in 10% neutral-buffered formalin at the animal facility. Samples were embedded in paraffin, sectioned, and transferred to slides for hematoxylin/eosin and immunohistochemical staining. A pathologist reviewed each case and delimited the region of interest, containing epithelial and stromal cells, for each specimen. A mouse TMA was prepared at the Tissue Core Facility at the Moffitt Cancer Center. The mouse TMA contained a total of 113 core samples, which included 10 uterine horns and 10 ovaries from both PBS- and HCQ-treated mice. As control specimens for the antibodies used, the TMA included mouse mammary tissue, liver, small intestine, and lymph nodes from a PBS-treated mouse. Lesions were analyzed from independent blocks. Slides were stained using a Leica Bond RX automated system (Leica Biosytems, Buffalo Grove, IL, USA) following the manufacturer's instructions with proprietary reagents. Slides were deparaffinized on an automated system with Dewax Solution (Leica Biosystems). The antigen retrieval method used for PR was enzymatic with Enzyme Solution 1 at 15 min (Leica), for vimentin and ER was heat induced with Epitope Retrieval Solution 1 at 20 min (Leica), for CK8 was heat induced with Epitope Retrieval Solution 2 at 10 min (Leica), and for LC3B was heat induced with Epitope Retrieval Solution 1 at 10 min (Leica Biosystems). All antibodies were diluted in Dako antibody diluent (Dako): PR (no. ab131486, 1 : 500; Abcam, Cambridge, MA, USA), vimentin (no. 5741 (D21H3), 1 : 100; Cell Signaling), ER *α* (no. ab32063 (E115), 1 : 200; Abcam), CK8 (no. ab53280 (EP1628Y), 1 : 200; Abcam), and LC3B (no. ab51520, 1 : 1500; Abcam) and incubated for 30 min. The Leica Bond Polymer Refine Detection System Leica Biosystems was used with a polymer incubation for 8 min. Hematoxylin was used as a counterstain, and slides were dehydrated and covered with a coverslip, following standard histological protocol.

### Analysis of murine peritoneal inflammatory molecules

After animals were killed, 1 ml of sterile PBS was injected into the peritoneal cavity, the abdominal area was gently massaged, and the fluid collected. The collected fluid was centrifuged at 1390 r.p.m. for 5 min at 4 °C and the resulting supernatant was then stored at −80 °C. Levels of chemokines and cytokines were analyzed using a MCYTOMAG-70K-PX32 (Millipore, Billerica, MA, USA) following the manufacturer's instructions. Briefly, 200 *μ*l of wash buffer was added to each well and incubated for 10 min at room temperature in a plate shaker. After incubation, the wash buffer was decanted and the plate was inverted and tapped on absorbent towel several times. Then, 25 *μ*l of assay buffer was added to each well, followed by 25 *μ*l of concentration standards, assay controls, or samples. The premixed bottle was vortexed and 25 *μ*l of the beads were added to each well. The plate was incubated overnight at 4 °C, and then protected from light. Then, the plate was incubated for 1 min on the hand-held magnet and the well content was gently decanted and tapped on absorbent pads. Each well was washed two times using 200 *μ*l of wash buffer, followed by the incubation on the hand-held magnet. Antibody detection solution was allowed to warm to room temperature, and then 25 *μ*l was added to each well and incubated for 1 h at room temperature on a plate shaker, protected from light. Next, 25 *μ*l of streptavidin-phycoerythrin was added to each well containing the detection antibodies and incubated for 30 min at room temperature protected from light on a plate shaker. After the incubation, the plate was washed two times as previously described and 150 *μ*l of sheath fluid was added to each well. The plate was analyzed using MAGPIX instrument and xPONENT software solutions, version 4.2 Luminex Corporation (Austin, TX, USA).

### Flow cytometry

The pellet obtained after centrifugation of the peritoneal fluid wash (see above) was used for macrophage staining. When necessary, red blood cell lysis was performed according to the manufacturer's protocol (eBioscience, San Diego, CA, USA). The cell pellets were resuspended in 1 ml cold PBS and transferred to flow cytometry tubes. Samples were centrifuged for 1 min at 1390 r.p.m. The supernatant was decanted and cells were resuspended in 100 *μ*l of PBS. Cells were blocked using 0.5 *μ*g of Mouse BD Fc, Block (no. 553141; BD Pharmingen, San Jose, CA, USA) for 5 min on ice. The cells were incubated in 0.4 *μ*g of APC rat anti-mouse CD11b clone M1/70 (no. 553312; BD Pharmingen) and anti- mPE-F4/80/EMR1 (no. FAB5580C; R&D Systems, Minneapolis, MN, USA) at room temperature for 30 min, protected from light. After incubation, 700 *μ*l of PBS was added to each tube and centrifuged for 1 min at 4 °C. The supernatant was decanted, and the cells were resuspended in 300 *μ*l in PBS and analyzed by flow cytometry.

### Transmission electron microscopy

Following induction of anesthesia, the abdominal cavity of the mice was opened to expose the uterine horns. Both uterine horns were removed and cut in cross-sections of 2–3 mm long pieces, which were then rinsed in 0.1 M phosphate buffer to remove excess blood, and placed in 2.5% glutaraldehyde in 0.1 M sodium phosphate buffer, pH 7.2, at 4 °C. The tissue was fixed in glutaraldehyde at 4 °C for 24 h. Following fixation, the tissue was rinsed in buffer, sliced into 1-mm-thick rings and postfixed in 1% osmium tetroxide at 4 °C for 2 h. Following buffer and distilled water rinses, the tissue was dehydrated through a graded series of acetone dilutions, cleared with propylene oxide, infiltrated overnight, embedded in LX 112 epoxy resin mix (Ladd Research, Williston, VT, USA), and polymerized at 70 °C. Entire cross-sections of the uterine horns were obtained at 0.25 –0.35 *μ*m thickness and 70–80 nm thickness, and stained with 1% toluidine blue stain (for light microscopy) or 8% uranyl acetate and Reynold's lead citrate (for electron microscopy), respectively. The endometrium of both control and experimental animals was observed and photographed using an FEI Morgagni TEM (FEI Company, Hillsboro, OR, USA) with an AMT ActiveVu camera (AMT, Woburn, MA, USA).

### Statistical analyses

All analyses were performed using GraphPad Prism software (version 6.04; GraphPad, La Jolla, CA, USA). To calculate the significance of the observed disorganization of epithelial cells in eutopic endometria from endometriosis-induced mice treated with HCQ (compared with those treated with PBS, as a control), we used the Fisher's exact test. All other statistical analyses were calculated using the nonparametric Student's *t*-test and error bars displayed represent standard errors of the mean (S.E.M.). Statistical significance was set at *P*⩽0.05 (*P⩽0.05, **P⩽0.01, ***P⩽0.001, and ****P⩽0.0001).

## Abbreviations

ATG: autophagy-related gene
BNIP3: BCL2/adenovirus E1B 19 kDa interacting protein
CK8: cytokeratin 8
EIF2AK3: eukaryotic translation initiation factor 2-*α* kinase 3
ER *α*: estrogen receptor-α
FBS: fetal bovine serum
GABARAPL1: GABA(A) receptor-associated protein like 1
G-CSF: granulocyte colony-stimulating factor
GI: gastrointestinal tract
HCQ: hydroxychloroquine
IGF1: insulin-like growth factor 1
IP-10: 10 kDa interferon-γ-induced protein
IRGM1: immunity-related GTPase family M1
ITS: insulin transferrin selenium
LC3B: microtubule-associated protein 1 light-chain 3*β*
PE: phosphatidylethanolamine
PBS: phosphate-buffered saline
PRKAA1: protein kinase AMP-activated, *α*1 catalytic subunit
PR: progesterone receptor
PTEN: phosphatase and tensin homolog
SQSTM1: Sequestosome 1
TEM: transmission electron microscopy
TMA: tissue microarray

## References

[bib1] Giudice LC, Kao LC. Endometriosis. Lancet 2004; 364: 1789–1799. 15541453 10.1016/S0140-6736(04)17403-5

[bib2] Bulun SE. Endometriosis. N Engl J Med 2009; 360: 268–279. 19144942 10.1056/NEJMra0804690

[bib3] Giudice LC. Clinical practice. Endometriosis. N Engl J Med 2010; 362: 2389–2398. 20573927 10.1056/NEJMcp1000274 PMC3108065

[bib4] Sourial S, Tempest N, Hapangama DK. Theories on the pathogenesis of endometriosis. Int J Reprod Med 2014; 2014: 179515. 25763392 10.1155/2014/179515 PMC4334056

[bib5] Sampson JA. Peritoneal endometriosis due to the menstrual dissemination of endometrial tissue into the peritoneal cavity. Am J Obstet Gynecol 1927; 14: 422–469.

[bib6] Baldi A, Campioni M, Signorile PG. Endometriosis: pathogenesis, diagnosis, therapy and association with cancer (review). Oncol Rep 2008; 19: 843–846. 18357365

[bib7] Vercellini P, Vigano P, Somigliana E, Fedele L. Endometriosis: pathogenesis and treatment. Nat Rev Endocrinol 2014; 10: 261–275. 24366116 10.1038/nrendo.2013.255

[bib8] Fung C, Lock R, Gao S, Salas E, Debnath J. Induction of autophagy during extracellular matrix detachment promotes cell survival. Mol Biol Cell 2008; 19: 797–806. 18094039 10.1091/mbc.E07-10-1092 PMC2262959

[bib9] Lamb CA, Yoshimori T, Tooze SA. The autophagosome: origins unknown, biogenesis complex. Nat Rev Mol Cell Biol 2013; 14: 759–774. 24201109 10.1038/nrm3696

[bib10] Feng Y, He D, Yao Z, Klionsky DJ. The machinery of macroautophagy. Cell Res 2014; 24: 24–41. 24366339 10.1038/cr.2013.168 PMC3879710

[bib11] Legakis JE, Yen WL, Klionsky DJ. A cycling protein complex required for selective autophagy. Autophagy 2007; 3: 422–432. 17426440 10.4161/auto.4129

[bib12] Pelch KE, Schroder AL, Kimball PA, Sharpe-Timms KL, Davis JW, Nagel SC. Aberrant gene expression profile in a mouse model of endometriosis mirrors that observed in women. Fertil Steril 2010; 93: 1615–1627, e1618. 19473656 10.1016/j.fertnstert.2009.03.086 PMC2904074

[bib13] Choi J, Jo M, Lee E, Kim HJ, Choi D. Differential induction of autophagy by mTOR is associated with abnormal apoptosis in ovarian endometriotic cysts. Mol Hum Reprod 2014; 20: 309–317. 24319109 10.1093/molehr/gat091

[bib14] Allavena G, Carrarelli P, Del Bello B, Luisi S, Petraglia F, Maellaro E. Autophagy is upregulated in ovarian endometriosis: a possible interplay with p53 and heme oxygenase-1. Fertil Steril 2015; 103: 1244–1251, e1241. 25772769 10.1016/j.fertnstert.2015.02.007

[bib15] Al-Bari MA. Chloroquine analogues in drug discovery: new directions of uses, mechanisms of actions and toxic manifestations from malaria to multifarious diseases. J Antimicrob Chemother 2015; 70: 1608–1621. 25693996 10.1093/jac/dkv018 PMC7537707

[bib16] Amaravadi RK, Lippincott-Schwartz J, Yin XM, Weiss WA, Takebe N, Timmer W et al. Principles and current strategies for targeting autophagy for cancer treatment. Clin Cancer Res 2011; 17: 654–666. 21325294 10.1158/1078-0432.CCR-10-2634 PMC3075808

[bib17] Calabretta B, Salomoni P. Inhibition of autophagy: a new strategy to enhance sensitivity of chronic myeloid leukemia stem cells to tyrosine kinase inhibitors. Leuk Lymphoma 2011; 52: 54–59. 21250825 10.3109/10428194.2010.546913 PMC7416844

[bib18] Bauckman KA, Haller E, Flores I, Nanjundan M. Iron modulates cell survival in a Ras- and MAPK-dependent manner in ovarian cells. Cell Death Dis 2013; 4: e592. 23598404 10.1038/cddis.2013.87 PMC3668627

[bib19] Somigliana E, Vigano P, Rossi G, Carinelli S, Vignali M, Panina-Bordignon P. Endometrial ability to implant in ectopic sites can be prevented by interleukin-12 in a murine model of endometriosis. Hum Reprod 1999; 14: 2944–2950. 10601076 10.1093/humrep/14.12.2944

[bib20] Mariani M, Vigano P, Gentilini D, Camisa B, Caporizzo E, Di Lucia P et al. The selective vitamin D receptor agonist, elocalcitol, reduces endometriosis development in a mouse model by inhibiting peritoneal inflammation. Hum Reprod 2012; 27: 2010–2019. 22588001 10.1093/humrep/des150

[bib21] McAfee Q, Zhang Z, Samanta A, Levi SM, Ma XH, Piao S et al. Autophagy inhibitor Lys05 has single-agent antitumor activity and reproduces the phenotype of a genetic autophagy deficiency. Proc Natl Acad Sci USA 2012; 109: 8253–8258. 22566612 10.1073/pnas.1118193109 PMC3361415

[bib22] Bacci M, Capobianco A, Monno A, Cottone L, Di Puppo F, Camisa B et al. Macrophages are alternatively activated in patients with endometriosis and required for growth and vascularization of lesions in a mouse model of disease. Am J Pathol 2009; 175: 547–556. 19574425 10.2353/ajpath.2009.081011 PMC2716955

[bib23] Jang CH, Choi JH, Byun MS, Jue DM. Chloroquine inhibits production of TNF-alpha, IL-1beta and IL-6 from lipopolysaccharide-stimulated human monocytes/macrophages by different modes. Rheumatology (Oxford) 2006; 45: 703–710. 16418198 10.1093/rheumatology/kei282

[bib24] Dinulescu DM, Ince TA, Quade BJ, Shafer SA, Crowley D, Jacks T. Role of K-ras and Pten in the development of mouse models of endometriosis and endometrioid ovarian cancer. Nat Med 2005; 11: 63–70. 15619626 10.1038/nm1173

[bib25] Matsuzaki S, Canis M, Pouly JL, Botchorishvili R, Dechelotte PJ, Mage G. Differential expression of genes in eutopic and ectopic endometrium from patients with ovarian endometriosis. Fertil Steril 2006; 86: 548–553. 16815388 10.1016/j.fertnstert.2006.02.093

[bib26] Meola J, Rosa e Silva JC, Dentillo DB, da Silva WA Jr., Veiga-Castelli LC, Bernardes LA et al. Differentially expressed genes in eutopic and ectopic endometrium of women with endometriosis. Fertil Steril 2010; 93: 1750–1773. 19200988 10.1016/j.fertnstert.2008.12.058

[bib27] Klionsky DJ, Abdalla FC, Abeliovich H, Abraham RT, Acevedo-Arozena A, Adeli K et al. Guidelines for the use and interpretation of assays for monitoring autophagy. Autophagy 2012; 8: 445–544. 22966490 10.4161/auto.19496 PMC3404883

[bib28] Colon-Diaz M, Baez-Vega P, Garcia M, Ruiz A, Monteiro JB, Fourquet J et al. HDAC1 and HDAC2 are differentially expressed in endometriosis. Reprod Sci 2012; 19: 483–492. 22344732 10.1177/1933719111432870 PMC3343094

[bib29] Lee SJ, Silverman E, Bargman JM. The role of antimalarial agents in the treatment of SLE and lupus nephritis. Nat Rev Nephrol 2011; 7: 718–729. 22009248 10.1038/nrneph.2011.150

[bib30] Nothnick WB. Treating endometriosis as an autoimmune disease. Fertil Steril 2001; 76: 223–231. 11476764 10.1016/s0015-0282(01)01878-7

[bib31] Nirgianakis K, Bersinger NA, McKinnon B, Kostov P, Imboden S, Mueller MD. Regression of the inflammatory microenvironment of the peritoneal cavity in women with endometriosis by GnRHa treatment. Eur J Obstet Gynecol Reprod Biol 2013; 170: 550–554. 23993133 10.1016/j.ejogrb.2013.08.010

[bib32] Sinaii N, Cleary SD, Ballweg ML, Nieman LK, Stratton P. High rates of autoimmune and endocrine disorders, fibromyalgia, chronic fatigue syndrome and atopic diseases among women with endometriosis: a survey analysis. Hum Reprod 2002; 17: 2715–2724. 12351553 10.1093/humrep/17.10.2715

[bib33] Kvaskoff M, Mu F, Terry KL, Harris HR, Poole EM, Farland L et al. Endometriosis: a high-risk population for major chronic diseases? Hum Reprod Update 2015; 21: 500–516. 25765863 10.1093/humupd/dmv013 PMC4463000

[bib34] Ben-Zvi I, Kivity S, Langevitz P, Shoenfeld Y. Hydroxychloroquine: from malaria to autoimmunity. Clin Rev Allergy Immunol 2012; 42: 145–153. 21221847 10.1007/s12016-010-8243-x PMC7091063

[bib35] von Adamek EV, Simoes MJ, Freitas V, Patriarca MT, Soares JM Jr., Baracat EC. Lysosomal evaluation of endometrioma capsule epithelium and endometrium of patients with or without endometriosis. Clin Exp Obstet Gynecol 2005; 32: 27–30. 15864932

[bib36] Lee IH, Kawai Y, Fergusson MM, Rovira II, Bishop AJ, Motoyama N et al. Atg7 modulates p53 activity to regulate cell cycle and survival during metabolic stress. Science 2012; 336: 225–228. 22499945 10.1126/science.1218395 PMC4721513

[bib37] Taub DD, Lloyd AR, Conlon K, Wang JM, Ortaldo JR, Harada A et al. Recombinant human interferon-inducible protein 10 is a chemoattractant for human monocytes and T lymphocytes and promotes T cell adhesion to endothelial cells. J Exp Med 1993; 177: 1809–1814. 8496693 10.1084/jem.177.6.1809 PMC2191047

[bib38] Strieter RM, Kunkel SL, Arenberg DA, Burdick MD, Polverini PJ. Interferon gamma-inducible protein 10 (IP-10), a member of the C-X-C chemokine family, is an inhibitor of angiogenesis. Biochem Biophys Res Commun 1995; 210: 51–57. 7537965 10.1006/bbrc.1995.1626

[bib39] Galleri L, Luisi S, Rotondi M, Romagnani P, Cobellis L, Serio M et al. Low serum and peritoneal fluid concentration of interferon-gamma-induced protein-10 (CXCL10) in women with endometriosis. Fertil Steril 2009; 91: 331–334. 18281042 10.1016/j.fertnstert.2007.11.075

[bib40] Mei J, Zhu XY, Jin LP, Duan ZL, Li DJ, Li MQ. Estrogen promotes the survival of human secretory phase endometrial stromal cells via CXCL12/CXCR4 up-regulation-mediated autophagy inhibition. Hum Reprod 2015; 30: 1677–1689. 25976655 10.1093/humrep/dev100

[bib41] Burney RO, Talbi S, Hamilton AE, Vo KC, Nyegaard M, Nezhat CR et al. Gene expression analysis of endometrium reveals progesterone resistance and candidate susceptibility genes in women with endometriosis. Endocrinology 2007; 148: 3814–3826. 17510236 10.1210/en.2006-1692

[bib42] Marino G, Uria JA, Puente XS, Quesada V, Bordallo J, Lopez-Otin C. Human autophagins, a family of cysteine proteinases potentially implicated in cell degradation by autophagy. J Biol Chem 2003; 278: 3671–3678. 12446702 10.1074/jbc.M208247200

[bib43] Geng J, Klionsky DJ. The Atg8 and Atg12 ubiquitin-like conjugation systems in macroautophagy. 'Protein modifications: beyond the usual suspects' review series. EMBO Rep 2008; 9: 859–864. 18704115 10.1038/embor.2008.163 PMC2529362

[bib44] Li M, Hou Y, Wang J, Chen X, Shao ZM, Yin XM. Kinetics comparisons of mammalian Atg4 homologues indicate selective preferences toward diverse Atg8 substrates. J Biol Chem 2011; 286: 7327–7338. 21177865 10.1074/jbc.M110.199059 PMC3044989

[bib45] Wordinger RJ, Dickey JF, Ellicott AR. Histochemical evaluation of the lipid droplet content of bovine oviductal and endometrial epithelial cells. J Reprod Fertil 1977; 49: 113–114. 556769 10.1530/jrf.0.0490113

[bib46] Shibata M, Yoshimura K, Tamura H, Ueno T, Nishimura T, Inoue T et al. LC3, a microtubule-associated protein1A/B light chain3, is involved in cytoplasmic lipid droplet formation. Biochem Biophys Res Commun 2010; 393: 274–279. 20132792 10.1016/j.bbrc.2010.01.121

[bib47] Taylor E, Williams C. Surgical treatment of endometriosis: location and patterns of disease at reoperation. Fertil Steril 2010; 93: 57–61. 19006792 10.1016/j.fertnstert.2008.09.085

[bib48] Laux-Biehlmann A, d'Hooghe T, Zollner TM. Menstruation pulls the trigger for inflammation and pain in endometriosis. Trends Pharmacol Sci 2015; 36: 270–276. 25899467 10.1016/j.tips.2015.03.004

[bib49] Hadfield R, Mardon H, Barlow D, Kennedy S. Delay in the diagnosis of endometriosis: a survey of women from the USA and the UK. Hum Reprod 1996; 11: 878–880. 8671344 10.1093/oxfordjournals.humrep.a019270

[bib50] Costedoat-Chalumeau N, Dunogue B, Leroux G, Morel N, Jallouli M, Le Guern V et al. A critical review of the effects of hydroxychloroquine and chloroquine on the eye. Clin Rev Allergy Immunol 2015; 49: 317–326. 25672591 10.1007/s12016-015-8469-8

[bib51] Janssen NM, Genta MS. The effects of immunosuppressive and anti-inflammatory medications on fertility, pregnancy, and lactation. Arch Intern Med 2000; 160: 610–619. 10724046 10.1001/archinte.160.5.610

[bib52] Bilotas MA, Olivares CN, Ricci AG, Baston JI, Bengochea TS, Meresman GF et al. Interplay between endometriosis and pregnancy in a mouse model. PLoS One 2015; 10: e0124900. 25915402 10.1371/journal.pone.0124900 PMC4411153

[bib53] Perecko T, Kassab RB, Vasicek O, Pekarova M, Jancinova V, Lojek A. The effects of chloroquine and hydroxychloroquine on nitric oxide production in RAW 264.7 and bone marrow-derived macrophages. Folia Biol (Praha) 2014; 60: 39–44. 25369339 10.14712/fb2014060S10039

[bib54] Smith DM, Patel S, Raffoul F, Haller E, Mills GB, Nanjundan M. Arsenic trioxide induces a beclin-1-independent autophagic pathway via modulation of SnoN/SkiL expression in ovarian carcinoma cells. Cell Death Differ 2010; 17: 1867–1881. 20508647 10.1038/cdd.2010.53 PMC2932795

