# Supplementary figures and images for: Effect of hydroxychloroquine and characterization of autophagy in a mouse model of endometriosis
Source: Cell Death Dis. 2016 Jan 14;7(1):e2059–. doi: 10.1038/cddis.2015.361 (PMC4816166; doi:10.1038/cddis.2015.361)

**Supplementary Table 1**


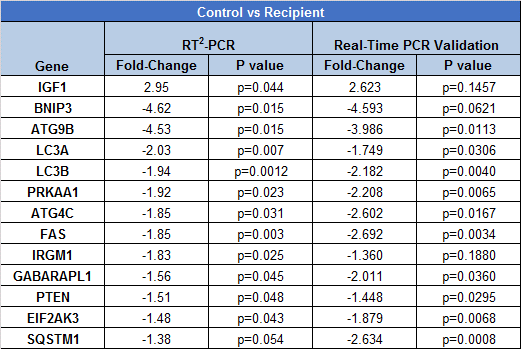

Supplement: Supplementary Table 1 [file cddis2015361x1.doc]

## Slide 1
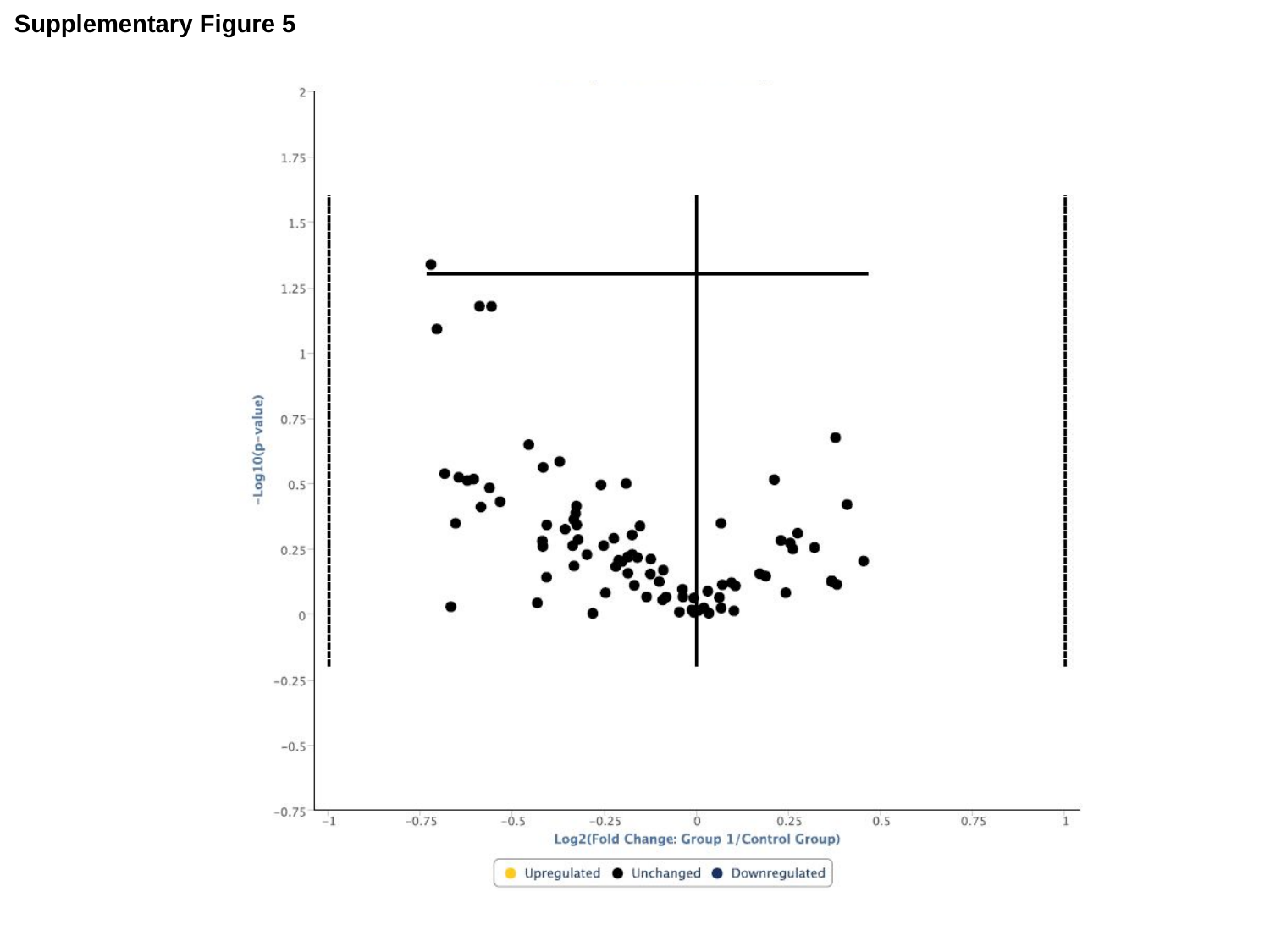

Supplementary Figure 5

Supplement: Supplementary Figure 6 [file cddis2015361x8.ppt]
