# Supplementary material for: Effect of hydroxychloroquine and characterization of autophagy in a mouse model of endometriosis
Source: Cell Death Dis. 2016 Jan 14;7(1):e2059–. doi: 10.1038/cddis.2015.361 (PMC4816166; doi:10.1038/cddis.2015.361)
Supplement: Supplementary Table 2 [file cddis2015361x2.doc]

**Supplementary Table 2**

| **Gene** | **Fold Change** | **P value** |
| --- | --- | --- |
| Beclin-1 | 2.20 | 0.0330 |
| p62 | 1.00 | NS |
| LC3B-I | 4.00 | 0.0185 |
| LC3B-II | 6.76 | 0.0364 |
| LC3A-I | 1.29 | NS |
| LC3A-II | 1.97 | 0.0135 |
| GABARAPL1 | 1.95 | 0.0334 |
| AMPKα | 0.86 | NS |
