# Supplementary material for: Effect of hydroxychloroquine and characterization of autophagy in a mouse model of endometriosis
Source: Cell Death Dis. 2016 Jan 14;7(1):e2059–. doi: 10.1038/cddis.2015.361 (PMC4816166; doi:10.1038/cddis.2015.361)
Supplement: Supplementary Figure 1 [file cddis2015361x3.ppt]

## Slide 1
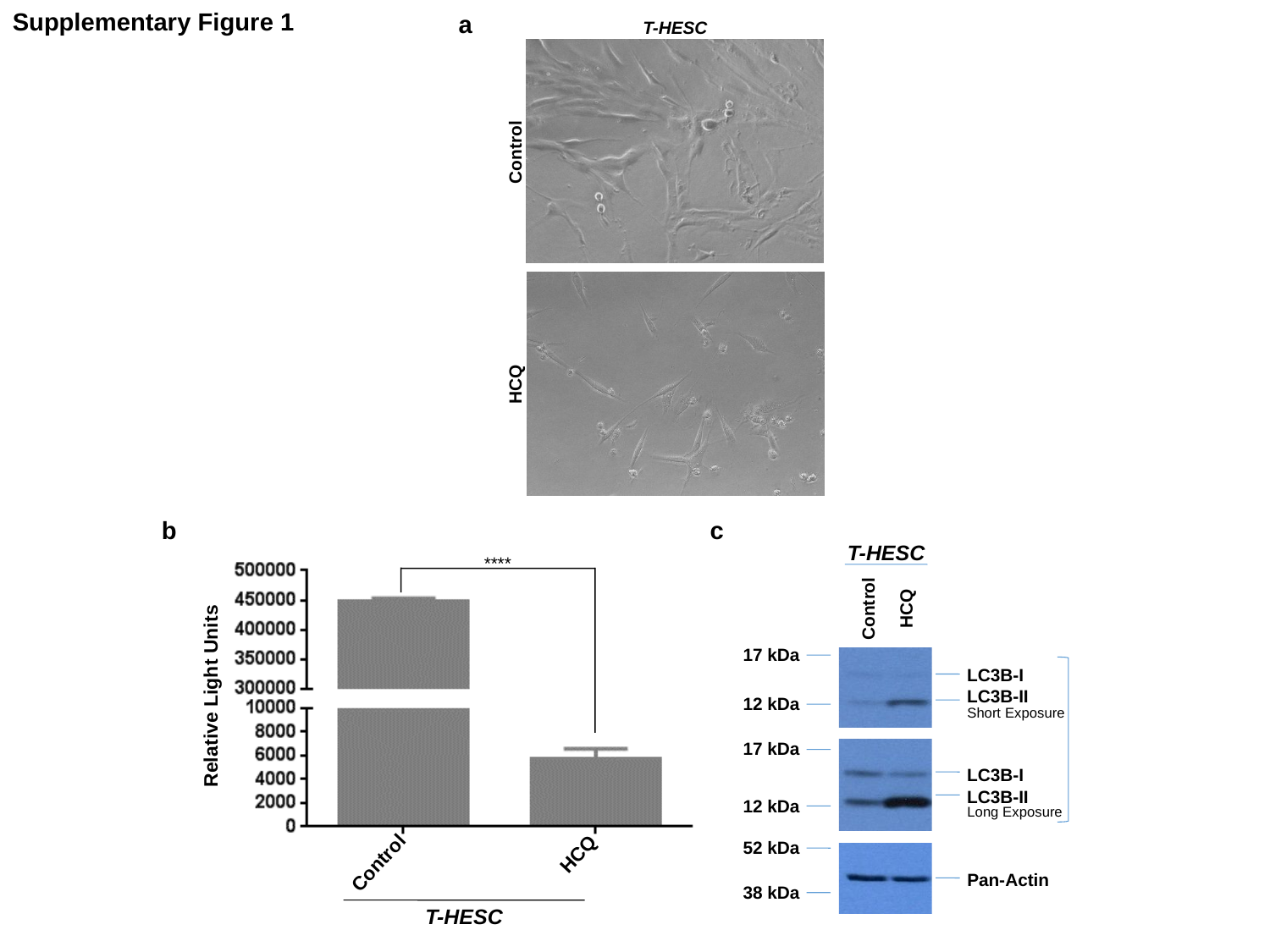

Supplementary Figure 1
a
T-HESC
Control
HCQ
b
c
T-HESC
Control
HCQ
17 kDa
LC3B-I
LC3B-II
12 kDa
Short Exposure
17 kDa
LC3B-I
LC3B-II
12 kDa
Long Exposure
52 kDa
Pan-Actin
38 kDa
****
Relative Light Units
HCQ
Control
T-HESC
