# Supplementary material for: Effect of hydroxychloroquine and characterization of autophagy in a mouse model of endometriosis
Source: Cell Death Dis. 2016 Jan 14;7(1):e2059–. doi: 10.1038/cddis.2015.361 (PMC4816166; doi:10.1038/cddis.2015.361)
Supplement: Supplementary Figure 2 [file cddis2015361x4.ppt]

## Slide 1
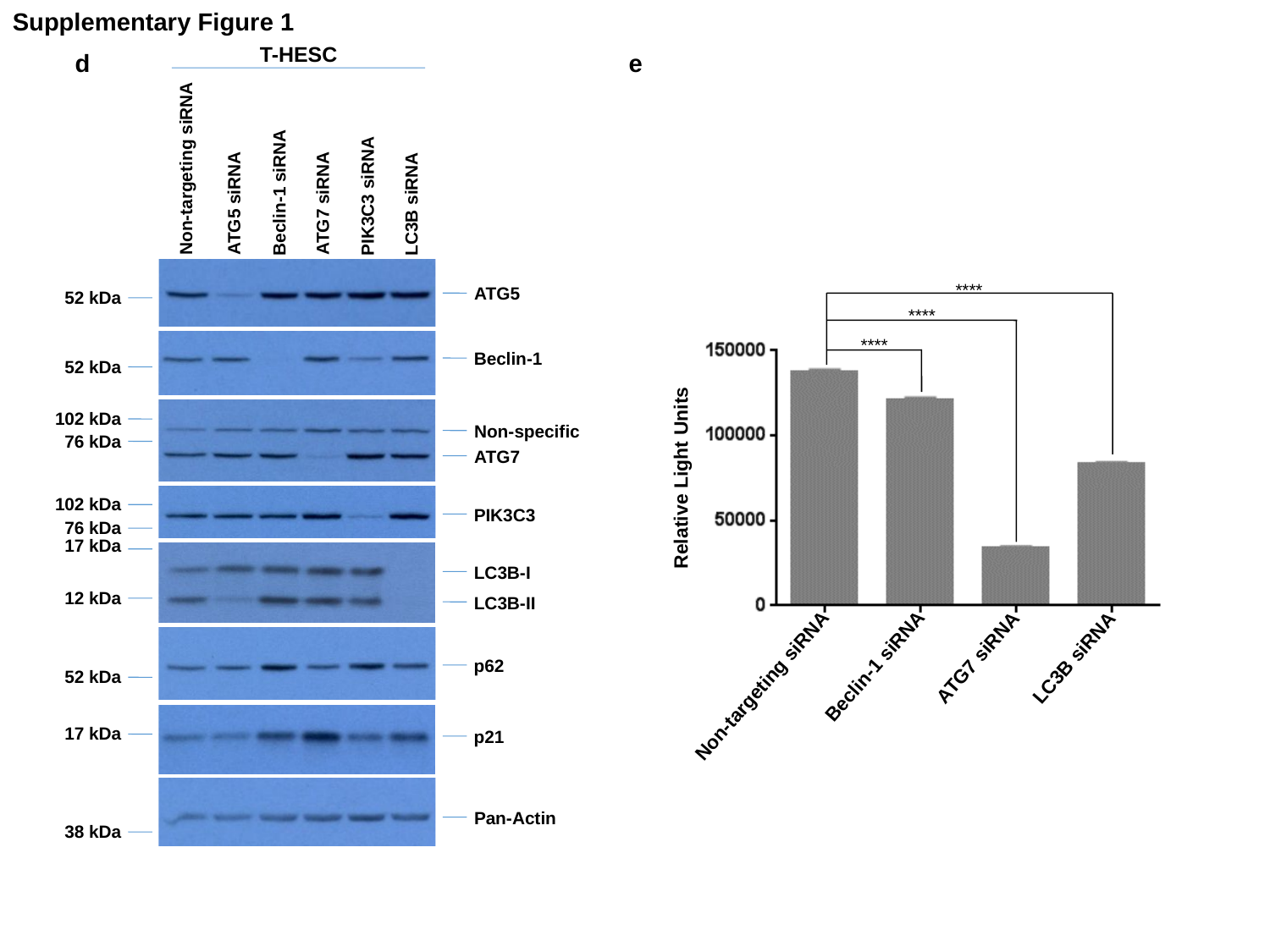

Supplementary Figure 1
T-HESC
Non-targeting siRNA
Beclin-1 siRNA
PIK3C3 siRNA
ATG5 siRNA
ATG7 siRNA
LC3B siRNA
ATG5
52 kDa
Beclin-1
52 kDa
102 kDa
Non-specific
76 kDa
ATG7
102 kDa
PIK3C3
76 kDa
17 kDa
LC3B-I
12 kDa
LC3B-II
p62
52 kDa
17 kDa
p21
Pan-Actin
38 kDa
d
e
****
****
****
Relative Light Units
ATG7 siRNA
LC3B siRNA
Beclin-1 siRNA
Non-targeting siRNA
