# Supplementary material for: Effect of hydroxychloroquine and characterization of autophagy in a mouse model of endometriosis
Source: Cell Death Dis. 2016 Jan 14;7(1):e2059–. doi: 10.1038/cddis.2015.361 (PMC4816166; doi:10.1038/cddis.2015.361)
Supplement: Supplementary Figure 3 [file cddis2015361x5.ppt]

## Slide 1
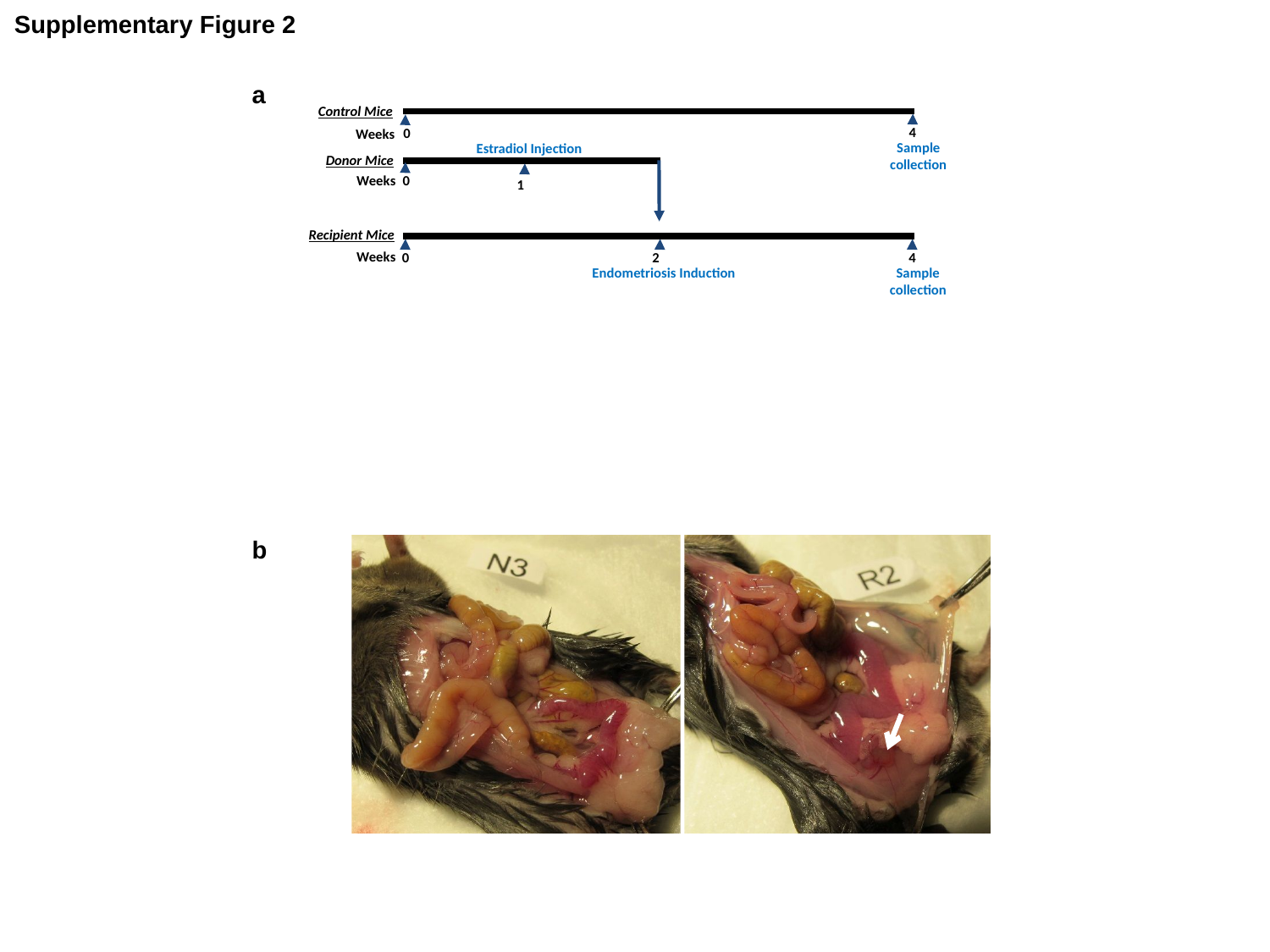

Supplementary Figure 2
a
Control Mice
4
 0
 Weeks
Sample collection
Estradiol Injection
Donor Mice
 0
 Weeks
1
Recipient Mice
 Weeks
 0
2
4
Endometriosis Induction
Sample collection
b
