# Supplementary material for: Effect of hydroxychloroquine and characterization of autophagy in a mouse model of endometriosis
Source: Cell Death Dis. 2016 Jan 14;7(1):e2059–. doi: 10.1038/cddis.2015.361 (PMC4816166; doi:10.1038/cddis.2015.361)
Supplement: Supplementary Figure 4 [file cddis2015361x6.ppt]

## Slide 1
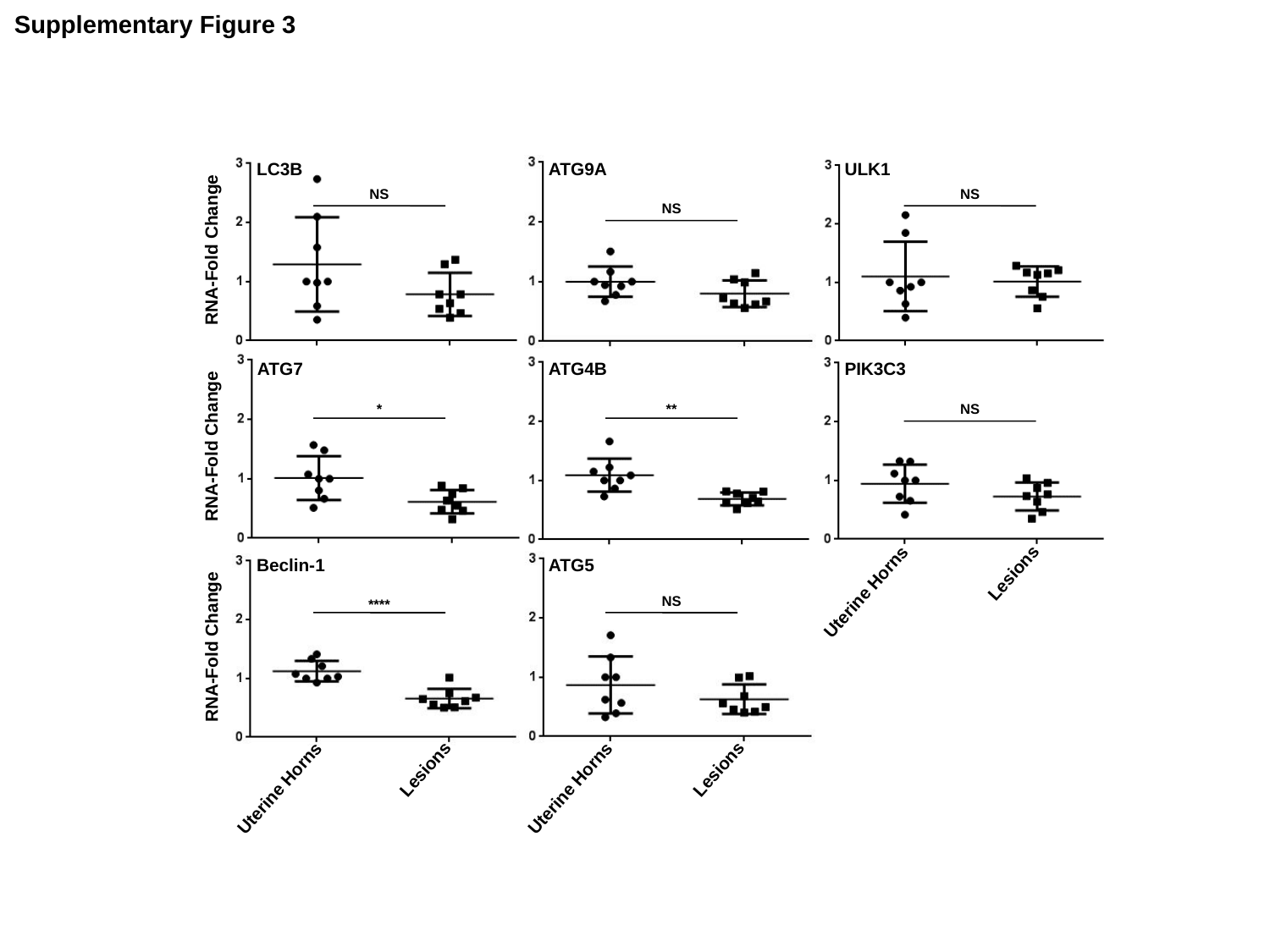

Supplementary Figure 3
LC3B
ATG9A
ULK1
NS
NS
NS
RNA-Fold Change
ATG7
ATG4B
PIK3C3
*
**
NS
RNA-Fold Change
Beclin-1
ATG5
Lesions
Uterine Horns
NS
****
RNA-Fold Change
Lesions
Lesions
Uterine Horns
Uterine Horns
