# Supplementary material for: Effect of hydroxychloroquine and characterization of autophagy in a mouse model of endometriosis
Source: Cell Death Dis. 2016 Jan 14;7(1):e2059–. doi: 10.1038/cddis.2015.361 (PMC4816166; doi:10.1038/cddis.2015.361)
Supplement: Supplementary Figure 5 [file cddis2015361x7.ppt]

## Slide 1
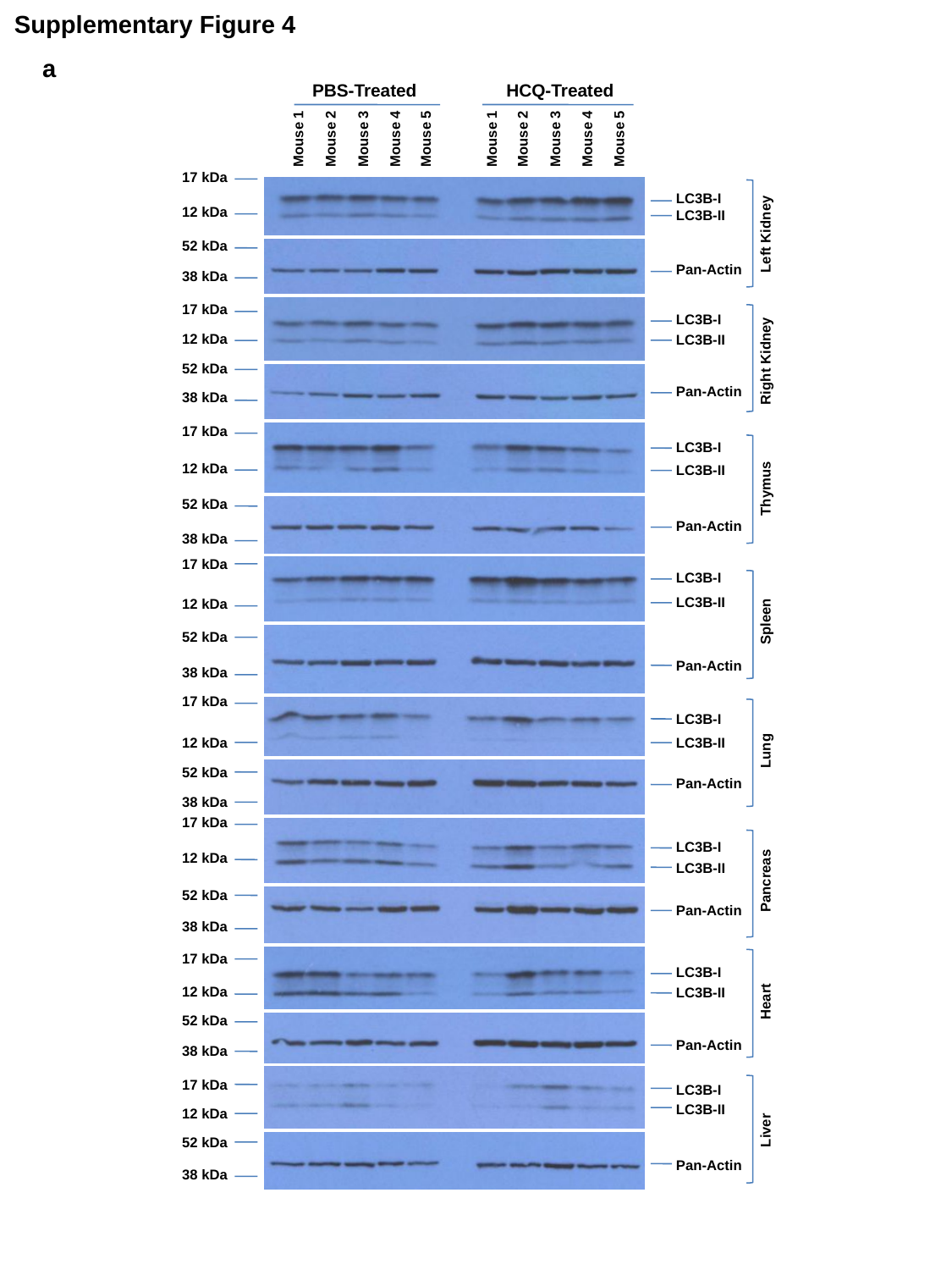

Supplementary Figure 4
a
PBS-Treated
HCQ-Treated
Mouse 1
Mouse 2
Mouse 3
Mouse 4
Mouse 5
Mouse 1
Mouse 2
Mouse 3
Mouse 4
Mouse 5
17 kDa
LC3B-I
12 kDa
LC3B-II
Left Kidney
52 kDa
Pan-Actin
38 kDa
17 kDa
LC3B-I
12 kDa
LC3B-II
Right Kidney
52 kDa
Pan-Actin
38 kDa
17 kDa
LC3B-I
12 kDa
LC3B-II
Thymus
52 kDa
Pan-Actin
38 kDa
17 kDa
LC3B-I
LC3B-II
12 kDa
Spleen
52 kDa
Pan-Actin
38 kDa
17 kDa
LC3B-I
12 kDa
LC3B-II
Lung
52 kDa
Pan-Actin
38 kDa
17 kDa
LC3B-I
12 kDa
LC3B-II
Pancreas
52 kDa
Pan-Actin
38 kDa
17 kDa
LC3B-I
12 kDa
LC3B-II
Heart
52 kDa
Pan-Actin
38 kDa
17 kDa
LC3B-I
LC3B-II
12 kDa
Liver
52 kDa
Pan-Actin
38 kDa

## Slide 2
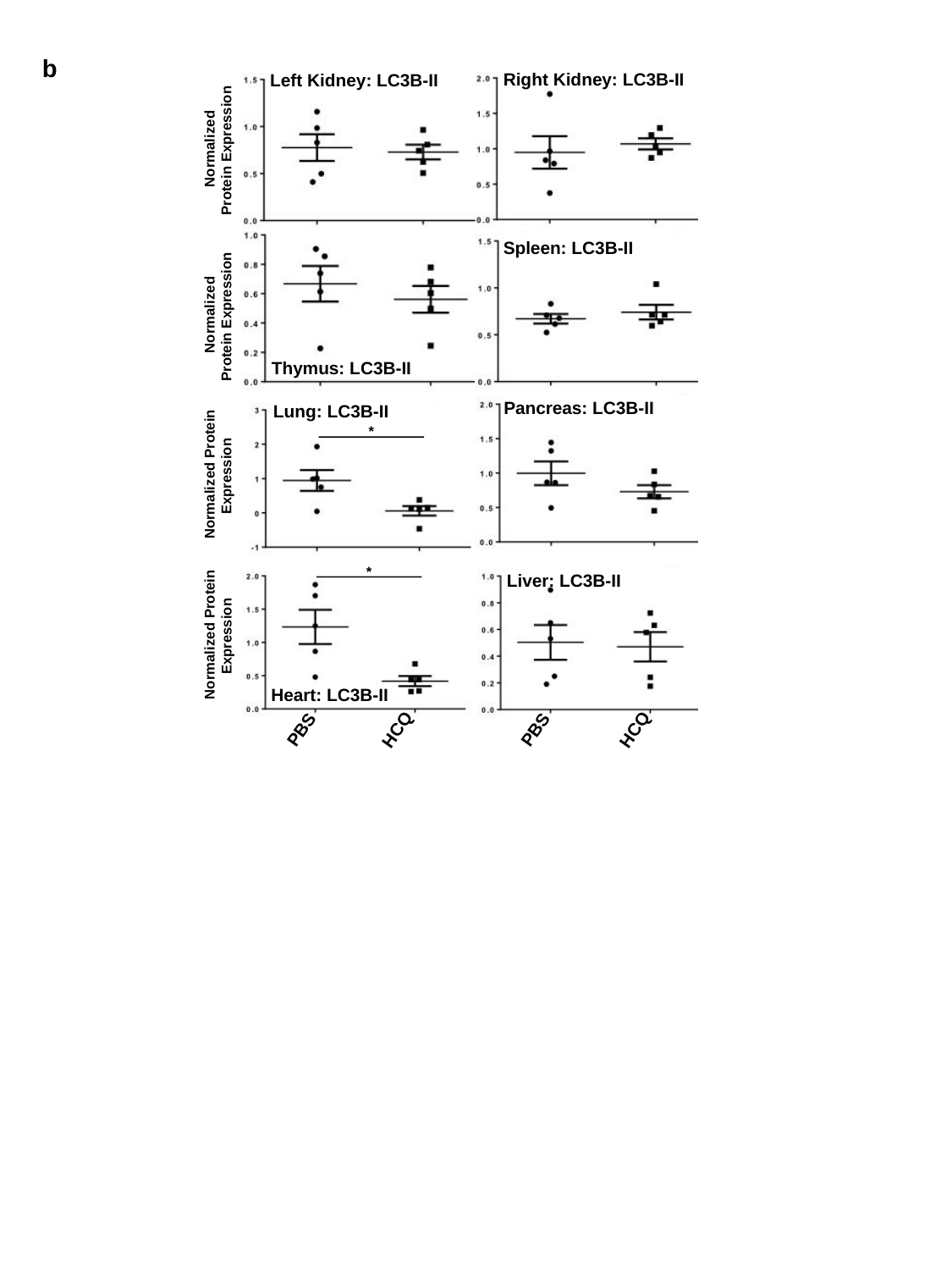

b
Right Kidney: LC3B-II
Left Kidney: LC3B-II
 Normalized Protein Expression
Spleen: LC3B-II
 Normalized Protein Expression
Thymus: LC3B-II
Pancreas: LC3B-II
Lung: LC3B-II
*
 Normalized Protein Expression
*
Liver: LC3B-II
 Normalized Protein Expression
Heart: LC3B-II
PBS
PBS
HCQ
HCQ
