# Supplementary material for: Effect of hydroxychloroquine and characterization of autophagy in a mouse model of endometriosis
Source: Cell Death Dis. 2016 Jan 14;7(1):e2059–. doi: 10.1038/cddis.2015.361 (PMC4816166; doi:10.1038/cddis.2015.361)
Supplement: Supplementary Figure 7 [file cddis2015361x9.ppt]

## Slide 1
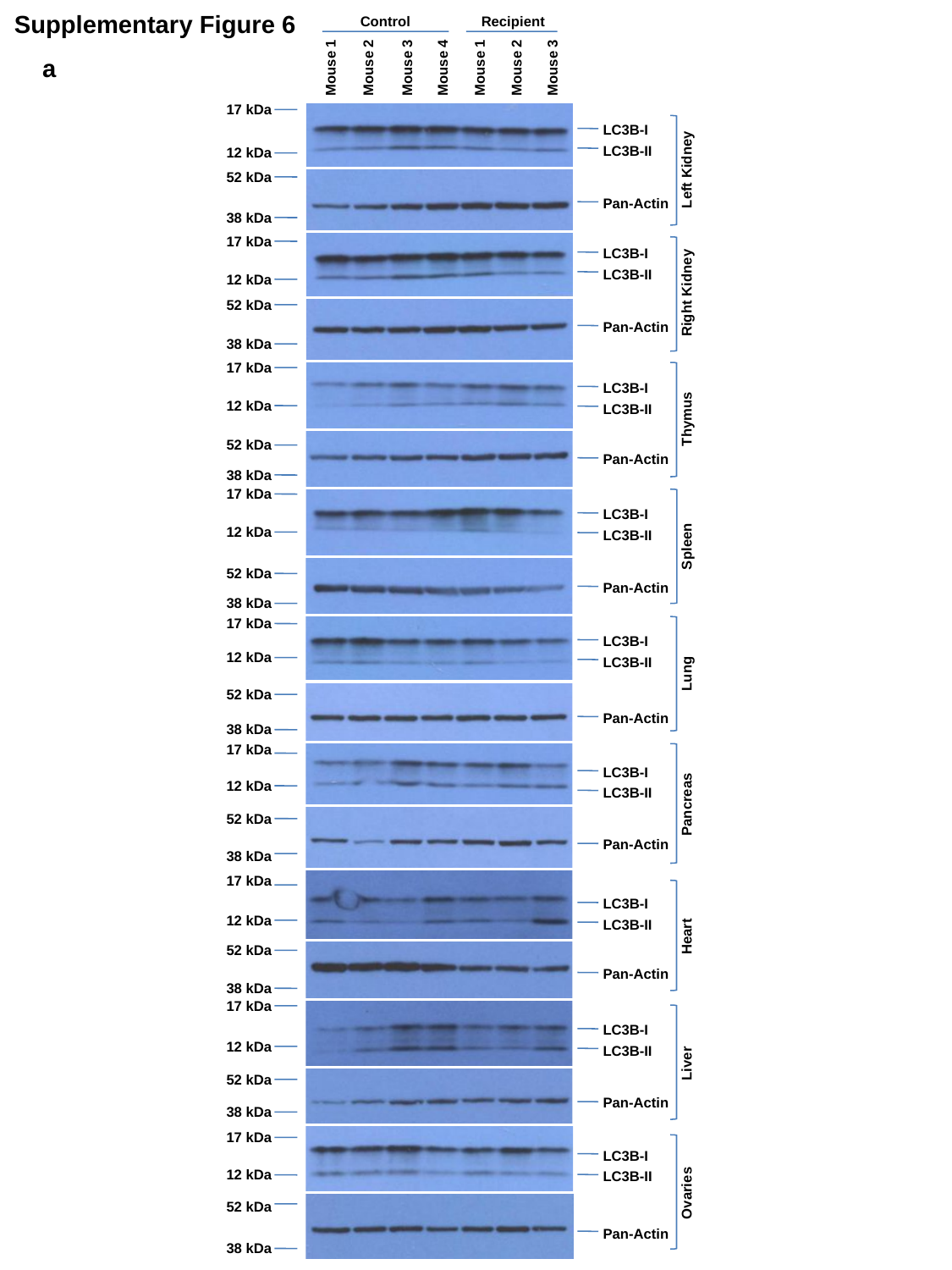

Control
Recipient
Mouse 1
Mouse 2
Mouse 3
Mouse 4
Mouse 1
Mouse 2
Mouse 3
17 kDa
LC3B-I
LC3B-II
12 kDa
Left Kidney
52 kDa
Pan-Actin
38 kDa
17 kDa
LC3B-I
LC3B-II
12 kDa
Right Kidney
52 kDa
Pan-Actin
38 kDa
17 kDa
LC3B-I
12 kDa
LC3B-II
Thymus
52 kDa
Pan-Actin
38 kDa
17 kDa
LC3B-I
12 kDa
LC3B-II
Spleen
52 kDa
Pan-Actin
38 kDa
17 kDa
LC3B-I
12 kDa
LC3B-II
Lung
52 kDa
Pan-Actin
38 kDa
17 kDa
LC3B-I
12 kDa
LC3B-II
Pancreas
52 kDa
Pan-Actin
38 kDa
17 kDa
LC3B-I
12 kDa
LC3B-II
Heart
52 kDa
Pan-Actin
38 kDa
17 kDa
LC3B-I
12 kDa
LC3B-II
Liver
52 kDa
Pan-Actin
38 kDa
17 kDa
LC3B-I
12 kDa
LC3B-II
Ovaries
52 kDa
Pan-Actin
38 kDa
Supplementary Figure 6
a

## Slide 2
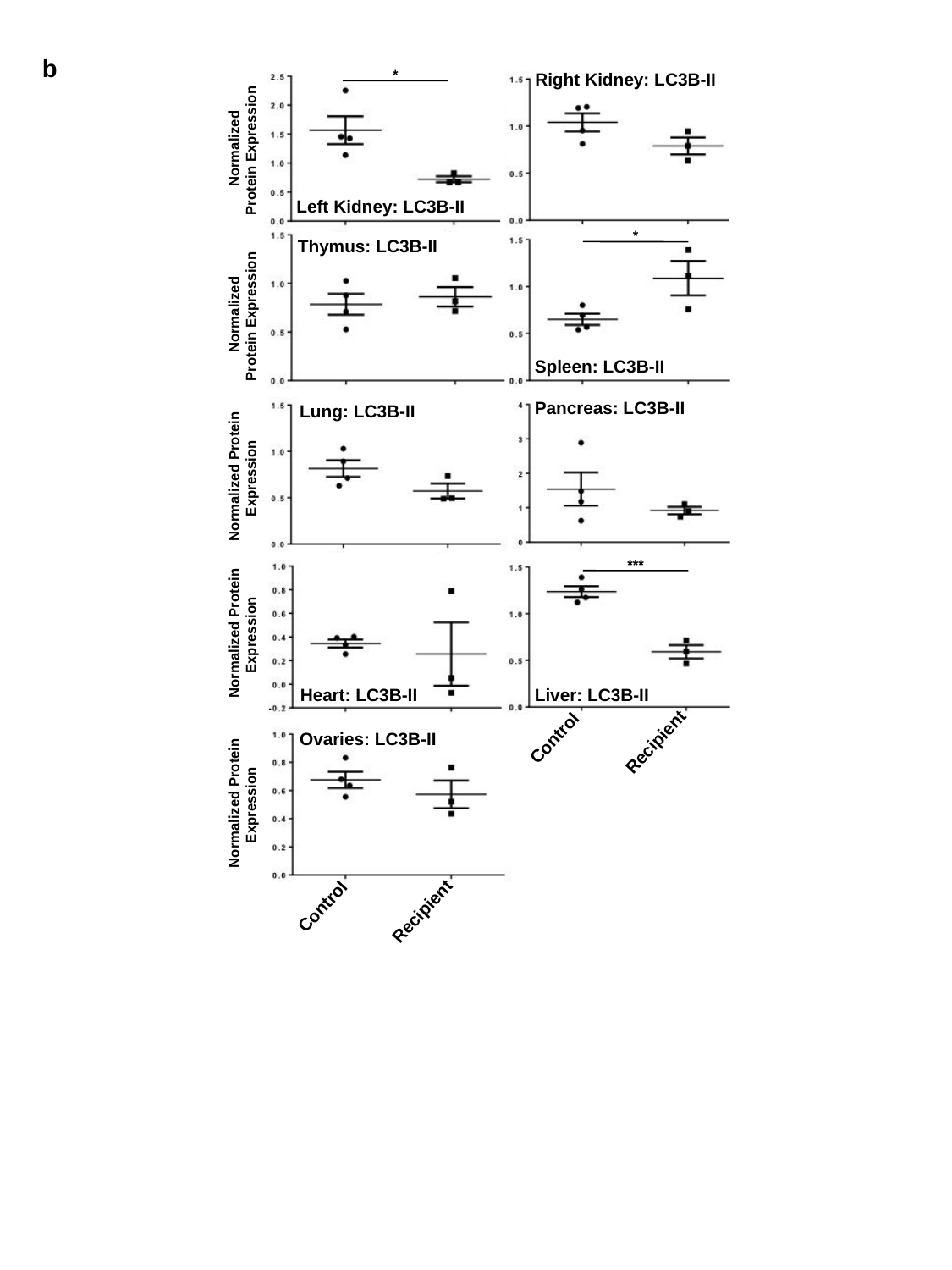

b
*
Right Kidney: LC3B-II
 Normalized Protein Expression
Left Kidney: LC3B-II
*
Thymus: LC3B-II
 Normalized Protein Expression
Spleen: LC3B-II
Pancreas: LC3B-II
Lung: LC3B-II
 Normalized Protein Expression
***
 Normalized Protein Expression
Heart: LC3B-II
Liver: LC3B-II
Control
Ovaries: LC3B-II
Recipient
 Normalized Protein Expression
Control
Recipient
