# Supplementary material for: Effect of hydroxychloroquine and characterization of autophagy in a mouse model of endometriosis
Source: Cell Death Dis. 2016 Jan 14;7(1):e2059–. doi: 10.1038/cddis.2015.361 (PMC4816166; doi:10.1038/cddis.2015.361)
Supplement: Supplementary Figure 8 [file cddis2015361x10.ppt]

## Slide 1
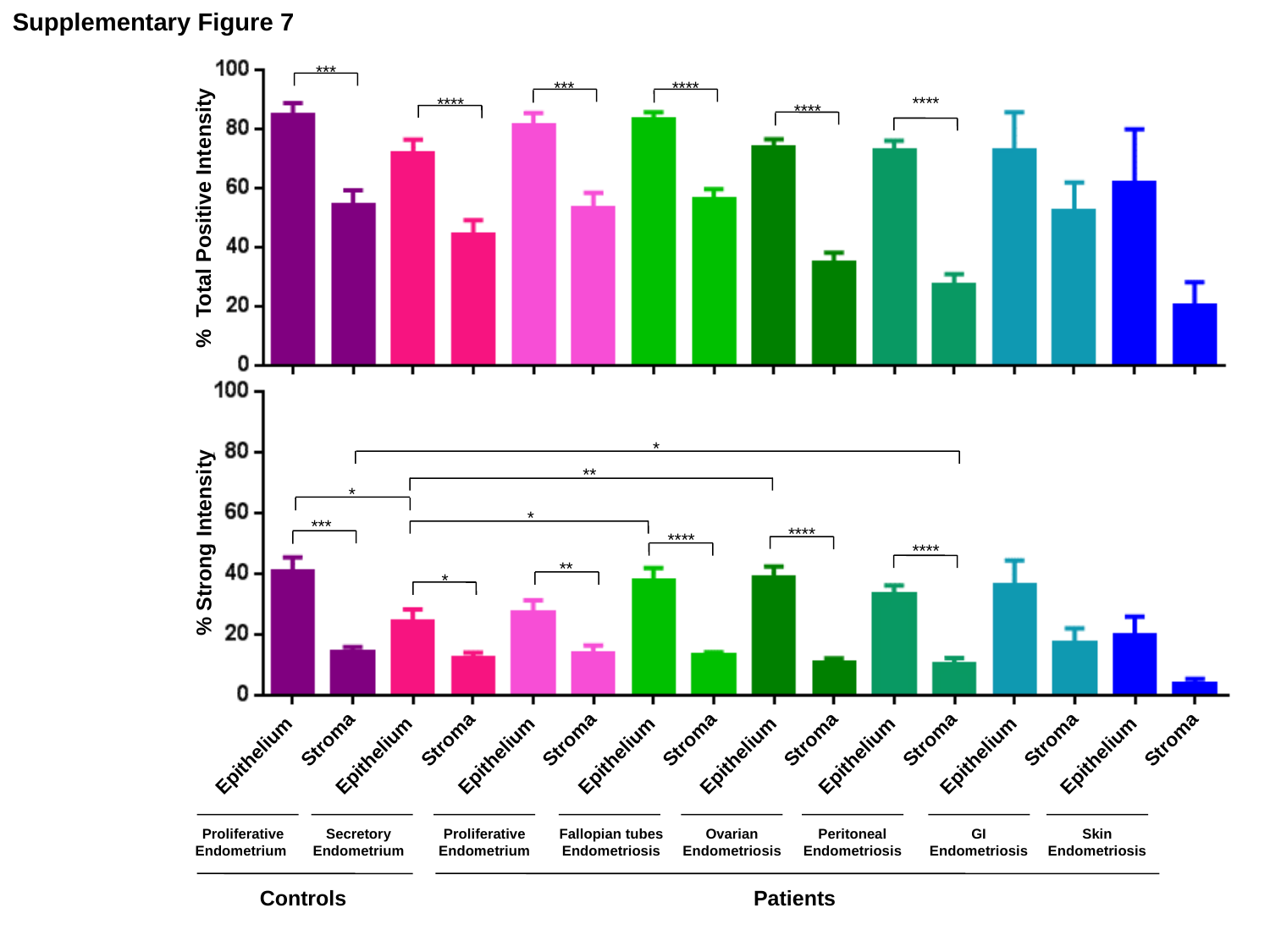

Supplementary Figure 7
***
***
****
****
****
****
% Total Positive Intensity
*
**
*
*
***
****
****
% Strong Intensity
****
**
*
Stroma
Stroma
Stroma
Stroma
Stroma
Stroma
Stroma
Stroma
Epithelium
Epithelium
Epithelium
Epithelium
Epithelium
Epithelium
Epithelium
Epithelium
Proliferative
Endometrium
Secretory Endometrium
Proliferative
Endometrium
Fallopian tubes Endometriosis
Ovarian Endometriosis
Peritoneal
Endometriosis
GI Endometriosis
Skin Endometriosis
Controls
Patients
