# Supplementary material for: Effect of hydroxychloroquine and characterization of autophagy in a mouse model of endometriosis
Source: Cell Death Dis. 2016 Jan 14;7(1):e2059–. doi: 10.1038/cddis.2015.361 (PMC4816166; doi:10.1038/cddis.2015.361)
Supplement: Supplementary Information [file cddis2015361x11.docx]

**Supplementary Information**

**Supplementary Figure 1** Hydroxychloroquine reduces human endometrial T-HESC cell survival. (**a**) Representative light micrographs of T-HESC cells treated with 25 μM HCQ for 18 h are shown. (**b**) Cell survival of T-HESC cells treated with 25 μM HCQ for five days was assessed by CellTiter-glo followed by luminescence measurements. Three independent experiments were performed. (**c**) Cell lysates from T-HESC cells treated with 25 μM HCQ for 18 h were analyzed by western blotting using the indicated antibodies. Three independent experiments were performed. (**d**) Cell lysates were collected from T-HESC cells treated with siRNA targeting ATG5, beclin-1, ATG7, PIK3C3, and LC3B. These samples were analyzed by western blotting using the indicated antibodies. (**e**) Cells were treated as described in Supplementary Figure 1d; cell viability was then assessed after three days following the second round of siRNA transfection, using CellTiter-glo.

**Supplementary Figure 2** Experimental outline for endometriosis induction in recipient mice. (**a**) The mouse strain used was C57BL/6. Mice in the control group were maintained without any treatment. Donor mice received an intraperitoneal injection of β-estradiol and two weeks later, mice were euthanized and the uterine horns were removed, minced, and injected into the peritoneal cavity of recipient mice. Two weeks after endometriosis induction, the recipient mice were euthanized and samples were collected. (**b**) Representative images of control (annotated as N (n=4), left panel) and recipient (annotated as R (n=3), right panel) mice. The peritoneal cavity was opened and images were captured after exposing the uterine horns. No lesions were present in any of the control mice and a total of 5 lesions were found amongst all three recipient mice. White arrow (right panel) indicates a lesion.

**Supplementary Figure 3** Autophagy markers are decreased in lesions compared to uterine horns from endometriosis-induced recipient mice. Uterine horns and lesions were collected from endometriosis-induced Balb/c mice and analyzed for autophagic gene expression. RNA-fold change was assessed by real-time PCR for the indicated autophagic markers. The line indicates the average values ± SEM.

**Supplementary Figure 4** (**a**) Protein expression of LC3B in a variety of tissues (kidneys, thymus, spleen, lungs, pancreas, heart, and liver) was analyzed by western blotting using Pan-Actin as a loading control. (**b**) Densitometric analyses of the presented western blots (right panels) are shown (presented as average ± SEM).

**Supplementary Figure 5** Gene expression comparison of autophagy-related genes between uterine horns of recipient (untreated) and PBS-treated recipient mice. As described in Figure 5a, RNA samples from uterine horns of recipient (untreated) and PBS-treated mice were used in the RT^2^-PCR array; a volcano plot is presented for these two treatment groups. Treatment with PBS did not significantly alter the analyzed genes by more than two-fold when compared to untreated recipient mice.

**Supplementary Figure 6** (**a**) Tissues (kidneys, thymus, spleen, lung, pancreas, hearts, liver, and ovaries) were collected from control and recipient mice and their homogenates analyzed for LC3B protein expression. (**b**) Densitometric analyses of the presented western blots (right panels) are shown (presented as average ± SEM).

**Supplementary Figure 7** LC3B analysis of the positive and strong positive staining intensity. The data obtained from the H-score system described in Figure 7 is presented as percent total positive intensity (upper panel) and percent strong intensity (lower panel) across the tissues described above.

**Supplementary Table 1** A comparison between the fold-change and statistical significance values from RT^2^-PCR array and TaqMan real-time PCR validation.

**Supplementary Table 2** The average fold-changes and p-values of the densitometric analyses of data shown in Figure 6A.
